# Human sperm TMEM95 binds eggs and facilitates membrane fusion

**DOI:** 10.1073/pnas.2207805119

**Published:** 2022-09-26

**Authors:** Shaogeng Tang, Yonggang Lu, Will M. Skinner, Mrinmoy Sanyal, Polina V. Lishko, Masahito Ikawa, Peter S. Kim

**Affiliations:** ^a^Department of Biochemistry, Stanford University School of Medicine, Stanford, CA 94305;; ^b^Sarafan ChEM-H, Stanford University, Stanford, CA 94305;; ^c^Immunology Frontier Research Center, Osaka University, Osaka 565-0871, Japan;; ^d^Department of Experimental Genome Research, Research Institute for Microbial Diseases, Osaka University, Osaka 565-0871, Japan;; ^e^Endocrinology Graduate Group, University of California, Berkeley, Berkeley, CA 94720;; ^f^Department of Molecular and Cell Biology, University of California, Berkeley, Berkeley, CA 94720;; ^g^Center for Reproductive Longevity and Equality, Buck Institute for Research on Aging, Novato, CA 94945;; ^h^Center for Infectious Disease Education and Research, Osaka University, Osaka 565-0871, Japan;; ^i^Laboratory of Reproductive Systems Biology, Institute of Medical Science, The University of Tokyo, Tokyo 108-8639, Japan;; ^j^Chan Zuckerberg Biohub, San Francisco, CA 94158

**Keywords:** TMEM95, membrane fusion, sperm–egg fusion, fertilization

## Abstract

*Tmem95* encodes a sperm acrosomal membrane protein, whose knockout has a male-specific sterility phenotype in mice. *Tmem95* knockout murine sperm can bind to, but do not fuse with, eggs. How TMEM95 plays a role in membrane fusion of sperm and eggs has remained elusive. Here, we utilize a sperm penetration assay as a model system to investigate the function of human TMEM95. We show that human TMEM95 binds to hamster egg membranes, providing evidence for a TMEM95 receptor on eggs. Using X-ray crystallography, we reveal an evolutionarily conserved, positively charged region of TMEM95 as a putative receptor-binding surface. Amino acid substitutions within this region of TMEM95 ablate egg-binding activity. We identify monoclonal antibodies against TMEM95 that reduce the number of human sperm fused with hamster eggs in sperm penetration assays. Strikingly, these antibodies do not block binding of sperm to eggs. Taken together, these results provide strong evidence for a specific, receptor-mediated interaction of sperm TMEM95 with eggs and suggest that this interaction may have a role in facilitating membrane fusion during fertilization.

## Significance

Membrane fusion of sperm and eggs is pivotal in sexual reproduction. *Tmem95* knockout mice produce sperm that can bind to, but do not fuse with, eggs. How TMEM95 facilitates membrane fusion was unknown. We show here that human TMEM95 binds eggs. Our crystal structure of TMEM95 suggests a region where this binding may occur. We develop monoclonal antibodies against TMEM95 that impair sperm-egg fusion but do not block sperm-egg binding. Thus, we propose that there is a receptor-mediated interaction of sperm TMEM95 with eggs, and that this interaction may have a direct role in membrane fusion. Our work suggests avenues for the identification of the TMEM95 egg receptor and the development of infertility treatments and contraceptives for humans.

Fertilization is a central event of sexual reproduction, but how sperm and eggs bind to and fuse with one another has been largely undefined. Sperm IZUMO1 (1) and egg JUNO (2) mediate the only known cell-surface interaction between mammalian gametes. Recent reports suggested that *TMEM95* (encoding transmembrane protein 95) mutant cattle (3, 4) and *Tmem95* mutant mice (5) exhibit impaired male fertility, and their sperm have defects in fusion with eggs; *Tmem95* knockout mice show male-specific sterility (6, 7). *Tmem95* knockout murine sperm, which have normal expression and localization of IZUMO1, can bind to, but do not fuse with, eggs (6, 7). *Tmem95* encodes a sperm acrosomal membrane protein, which relocalizes to the equatorial segment of the sperm head (3, 7) where membrane fusion with the egg takes place (8, 9). These observations shed light on a potential role of TMEM95 in sperm-egg membrane fusion.

Humans also express *TMEM95* transcripts (10). In this study, we utilized the sperm penetration assay (11), a clinical laboratory test that evaluates fusion of human sperm with eggs from Syrian golden hamsters (*Mesocricetus auratus*), as a model system. TMEM95 is a type I single-pass transmembrane protein (3, 6, 7). Motivated by a hypothesis that the ectodomain of TMEM95 binds to eggs through a specific, membrane-bound receptor on eggs, we found that a bivalent TMEM95 ectodomain protein binds hamster eggs, providing direct evidence for a TMEM95 receptor on eggs. The 1.5 Å-resolution X-ray crystal structure of TMEM95 we describe here reveals an evolutionarily conserved region of the protein with a positively charged surface. Amino acid substitutions within this region of TMEM95 ablate egg binding. We speculate that this region serves as an egg-receptor binding site for TMEM95.

We also found that human TMEM95 plays a role in membrane fusion. After generating two monoclonal antibodies that bind to different epitopes of TMEM95, we observed that neither antibody blocks binding of human sperm to hamster eggs, but both could inhibit membrane fusion of sperm with eggs. Taken together, our results provide evidence for a specific, receptor-mediated interaction of human sperm TMEM95 with eggs and inform strategies for the identification of this receptor. We propose that the interaction of TMEM95 with eggs facilitates membrane fusion of human sperm and eggs.

## Results

### A Bivalent TMEM95 Protein Binds Hamster Eggs.

**Fig. 1.**
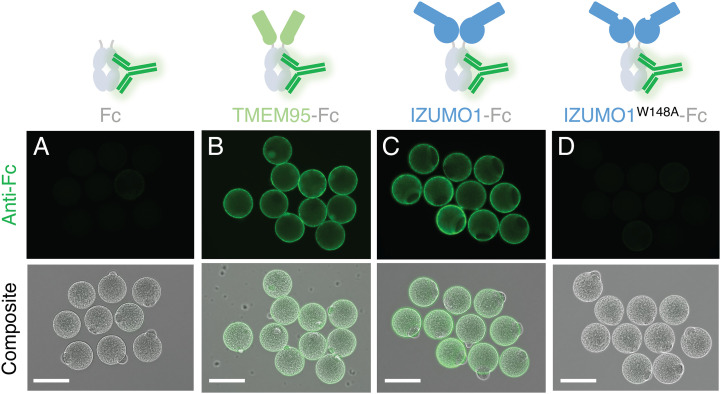
TMEM95-Fc binds eggs. Schematics of the Fc-fusion protein with a fluorescence-conjugated anti-Fc antibody. Immunofluorescence (*Upper*) and differential interference contrast composite images (*Lower*) of zona-free hamster eggs with 200 nM of (*A*) Fc, (*B*) TMEM95-Fc, (*C*) IZUMO1-Fc, or (*D*) IZUMO1^W148A^-Fc. Green fluorescence was conferred by a DyLight 488-conjugated anti-Fc antibody. (Scale bars, 100 μm). TMEM95-Fc and IZUMO1-Fc bind zona-free hamster eggs (*SI Appendix*, Fig. S1).

We hypothesized that the ectodomain of TMEM95 mediates a cell-surface interaction of sperm with eggs. To monitor the interaction between TMEM95 and eggs, we designed and produced TMEM95-Fc, a fusion protein of the ectodomain of human TMEM95 and the fragment crystallizable region of human immunoglobulin G1 (IgG1) (*SI Appendix*, Fig. S1*A*). TMEM95-Fc contains two copies of the TMEM95 ectodomain (Fig. 1*B*) and the Fc confers increased avidity for binding over monomeric TMEM95. Given that human sperm can fuse with eggs from Syrian golden hamsters (11, 12), we incubated the Fc or TMEM95-Fc proteins with hamster eggs, whose surrounding zona pellucida and cumulus cells were removed. Using a fluorescently labeled anti-Fc antibody, we detected binding to the hamster egg surface only with TMEM95-Fc, not Fc alone (Fig. 1 *A* and *B* and *SI Appendix*, Fig. S1*F*). We did not observe binding of TMEM95-Fc to murine eggs (*SI Appendix*, Fig. S1*B*). To confirm that our labeling approach can also detect known protein-protein interactions of sperm with eggs, we next surveyed IZUMO1-Fc on hamster eggs (13), a fusion protein of human sperm IZUMO1 (1) ectodomain with Fc. While IZUMO1-Fc binds eggs, the IZUMO1^W148A^-Fc variant does not (Fig. 1 *C* and *D*). The substitution of W148A ablates the interaction of IZUMO1 with JUNO (*SI Appendix*, Fig. S1 *C*–*I*) (14, 15), the egg receptor of IZUMO1 (2). Our results show that TMEM95 binds egg plasma membranes and suggest the presence of a receptor for TMEM95 on eggs.

### The Structure of TMEM95 Is Homologous to that of the N Terminus of IZUMO1.

**Fig. 2.**
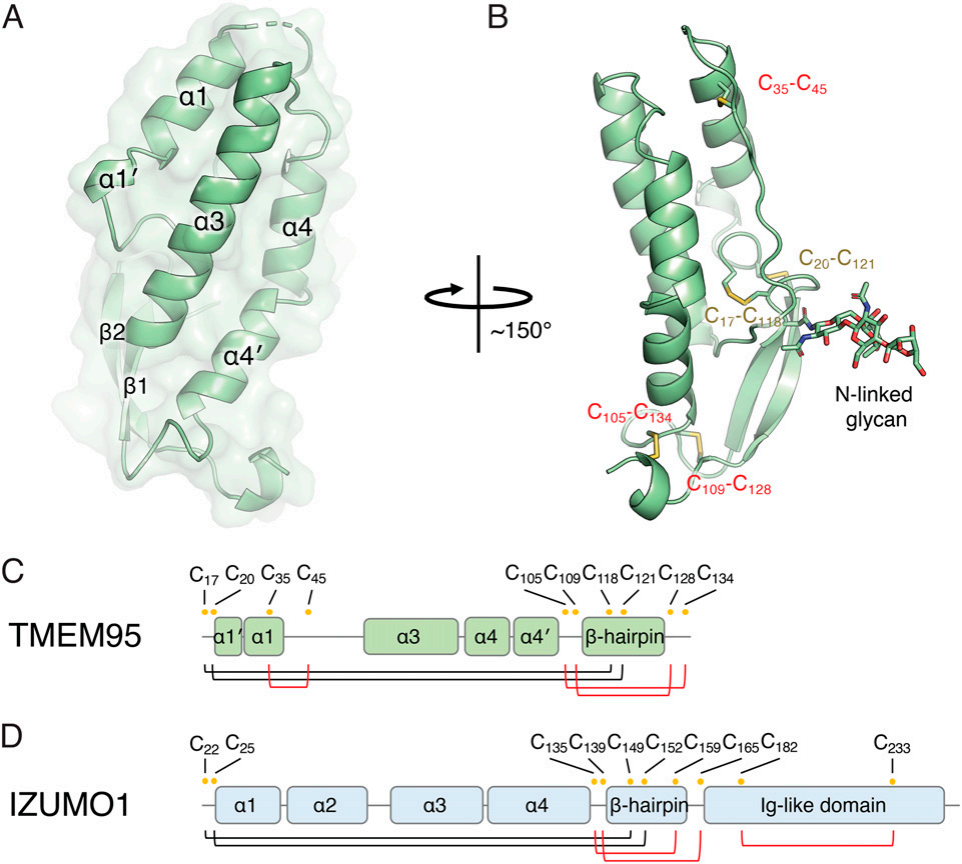
The structure of TMEM95 is homologous to IZUMO1. (*A*) Overlay of ribbon and space-filling diagrams of TMEM95 with structural elements labeled. (*B*) Ribbon diagram of TMEM95 with disulfide linkages labeled in yellow texts (same in IZUMO1) or red texts (different in IZUMO1). Domain organizations of (*C*) TMEM95 and (*D*) IZUMO1 with cysteine positions labeled as yellow dots and disulfide linked in black (same in TMEM95 and IZUMO1) or red lines (different between TMEM95 and IZUMO1). TMEM95 shows homology to the N terminus of IZUMO1 (*SI Appendix*, Fig. S2).

To understand how TMEM95 binds eggs, we determined a crystal structure of the TMEM95 ectodomain to 1.5 Å resolution using multiwavelength anomalous X-ray diffraction (Fig. 2*A* and *SI Appendix*, Fig. S2*A* and Table S1) (16). TMEM95 adopts an elongated rod shape, comprised of an N-terminal α-helical bundle (residues 17 to 110) and a C-terminal β-hairpin region (residues 111 to 135) (Fig. 2*C*). TMEM95 shows homology to the N terminus of IZUMO1 (14, 15) with a C_α_ root-mean-square deviation of 7.2 Å and to the N terminus of SPACA6 (17). Unlike IZUMO1 and SPACA6, TMEM95 does not have an immunoglobulin-like domain at the C terminus (Fig. 2*D*). The helical bundle of TMEM95 has three helices (α1, α3, and α4) and a coil (loop 2) that are arranged in an anti-parallel manner (α1-loop 2 and α3-α4). TMEM95 has three unique disulfide bonds: C35-C45 between α1 and loop 2 (*SI Appendix*, Fig. S2*B*), and C105-C134 and C109-C128 adjacent to the β-hairpin (Fig. 2 *B*–*D*).

JUNO does not act as an egg receptor for TMEM95 (6, 7). A conserved *N*-linked glycan in the β-hairpin of TMEM95 (*SI Appendix*, Fig. S2 *E* and *J*) could cause a clash if TMEM95 were to make a contact similar to that of IZUMO1 with JUNO (*SI Appendix*, Fig. S2 *C* and *D*). However, even if this glycan is removed by the treatment with *N*-glycosidase PNGaseF, TMEM95-Fc binds egg plasma membranes but does not bind JUNO (*SI Appendix*, Fig. S2 *F*–*I*).

### A Conserved Surface of TMEM95 Is a Putative Receptor-Binding Site.

**Fig. 3.**
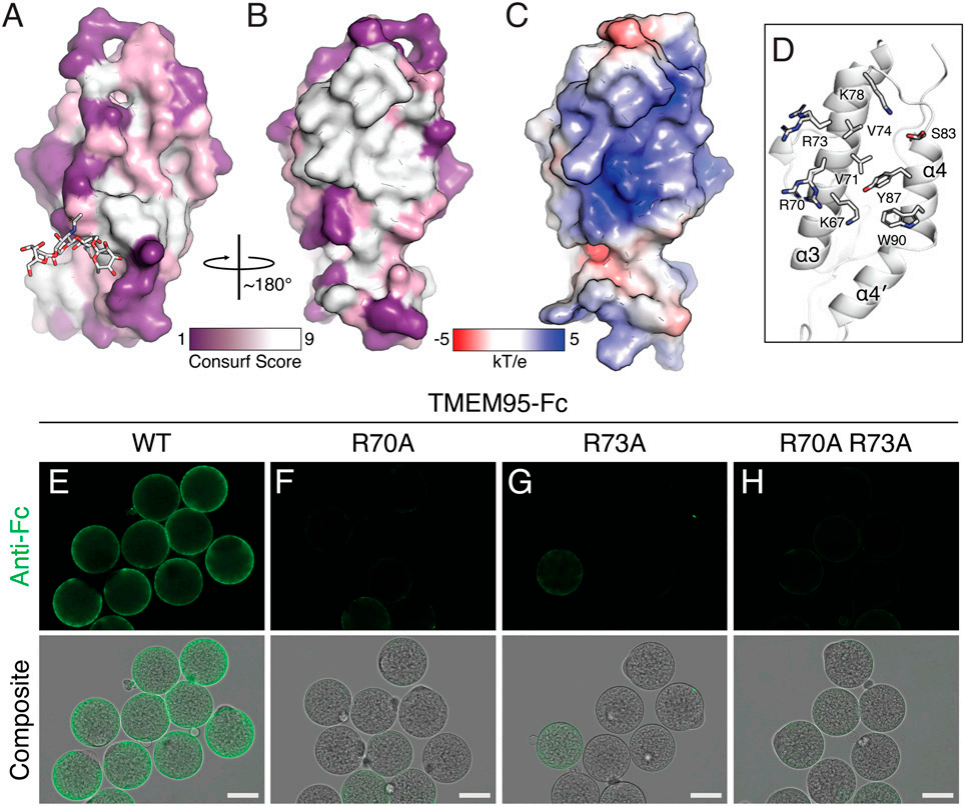
A conserved area of TMEM95 is a putative receptor binding site. (*A*, *B*) Space-filling *CONSURF* models (30) of TMEM95 with ∼180° rotation with purple representing variable and white representing conserved in TMEM95 orthologs. (*C*) Space-filling model of electrostatic surface potential generated by APBS (Adaptive Poisson-Boltzmann Solver) with blue representing positively charged and red representing negatively charged. (*D*) Ribbon diagram of the conserved area of TMEM95 showing the side chains of surface-exposed residues. (*E*–*H*) Immuno-fluorescence (*Upper*) and differential interference contrast composite images (*Lower*) of zona-free hamster eggs with 200 nM of (*E*) TMEM95^WT^-Fc, (*F*) TMEM95^R70A^-Fc, (*G*) TMEM95^R73A^-Fc, and (*H*) TMEM95^R70A R73A^-Fc. WT, wild type. Green fluorescence by a DyLight 488-conjugated anti-Fc antibody. (Scale bars, 50 μm). Substitutions of the conserved arginine residues on the identified surface of TMEM95 ablate egg-binding activities (*SI Appendix*, Fig. S3).

To gain further insights into the TMEM95 interaction with eggs, we analyzed the protein sequences of TMEM95 orthologs and mapped the degree of conservation for each amino acid onto the structure of TMEM95. We found that the area surrounding the *N*-glycan is variable (Fig. 3*A*), while the opposite side harbors a conserved (Fig. 3*B*), positively charged surface (Fig. 3*C*).

To examine whether the conserved, charged surface is critical for binding of TMEM95 to eggs, we produced TMEM95-Fc proteins that carry amino acid substitutions of arginine residues (Fig. 3*D* and *SI Appendix*, Fig. S3 *A* and *B*). These TMEM95 variants have melting temperatures comparable to that of the wild-type TMEM95-Fc protein (*SI Appendix*, Fig. S3*C*). When incubated with hamster eggs, the R70A, R73A, and R70A R73A TMEM95-Fc variants showed drastically reduced egg-binding activities compared to the wild-type (Fig. 3 *E*–*H* and *SI Appendix*, Fig. S3*D*). Our data suggest that the identified evolutionarily conserved, positively charged surface of TMEM95 may function as a receptor-binding site.

### Monoclonal Antibodies Detect TMEM95 in Human Sperm.

**Fig. 4.**
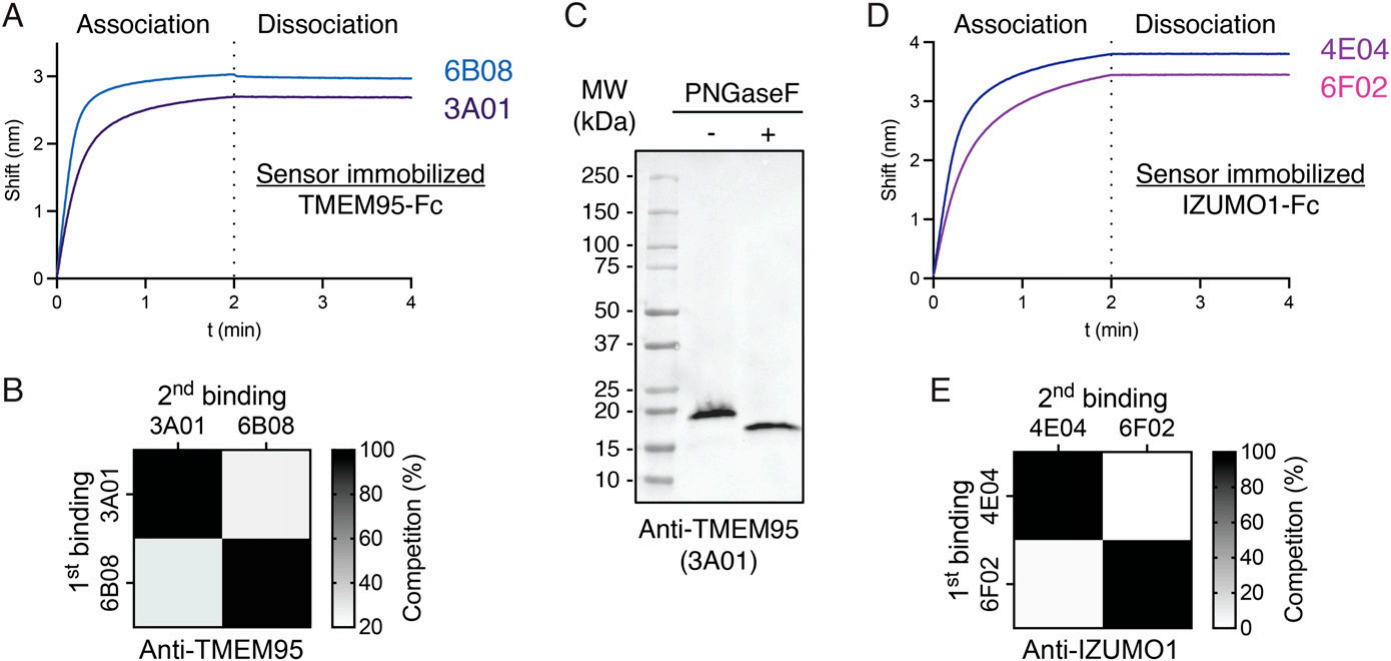
Antibodies detect the expression of TMEM95 in human sperm. (*A*, *D*) Biolayer interferometric traces of sensor immobilized (*A*) TMEM95-Fc binding to 200 nM of TMEM95 antibodies 3A01 IgG and 6B08 IgG or (*D*) IZUMO1-Fc binding to IZUMO1 antibodies 4E04 IgG and 6F02 IgG, with association for 2 min and dissociation for 2 min. (*B*, *E*) Summary in a heat map of antibody competition (*B*) of 3A01 IgG and 6B08 IgG to sensor immobilized TMEM95-Fc and (*E*) of 4E04 IgG and 6F02 IgG to sensor immobilized IZUMO1-Fc. (*C*) Human sperm lysates without or with PNGaseF treatments. Western blots were performed using non-heat-denatured, nonreduced sperm lysates by a primary antibody of 10 μg/mL anti-TMEM95 3A01 IgG, and a secondary horseradish peroxidase (HRP)-conjugated anti-mouse antibody. TMEM95 is expressed and *N*-linked glycosylated in human sperm (*SI Appendix*, Figs. S4 and S5).

To generate reagents to investigate the functions of TMEM95 in human sperm, we immunized mice with the TMEM95 ectodomain (*SI Appendix*, Fig. S4 *A*–*C*) and generated hybridoma cell lines that produce TMEM95 ectodomain-specific monoclonal antibodies, 3A01 and 6B08 (*SI Appendix*, Table S2). We used biolayer interferometry to assess the binding of the antibodies to TMEM95 (Fig. 4*A*) and found that 3A01 and 6B08 bind TMEM95 via two noncompeting epitopes (Fig. 4*B*) with association constants of 1.4 nM and 1.3 nM, respectively (*SI Appendix*, Fig. S4 *D* and *E*). The binding of either 3A01 or 6B08 to TMEM95-Fc does not inhibit its binding to the eggs (*SI Appendix*, Fig. S4*G*). 3A01 and 6B08 bind similarly to TMEM95-Fc and the R70A and R73A TMEM95-Fc variants (*SI Appendix*, Fig. S4*H*). These results suggest that the 3A01 and 6B08 antibodies against TMEM95 do not compete for binding of TMEM95 with its egg receptor.

We next performed Western blotting using the TMEM95 antibodies to probe whole cell lysates of human sperm and each could detect a band of ∼20 kDa (*SI Appendix*, Fig. S4*F*), the expected molecular weight of TMEM95. To investigate whether TMEM95 is *N*-linked glycosylated, we treated the human sperm lysate with PNGaseF and observed a shift in size to ∼17.5 kDa (Fig. 4*C*), consistent with the loss of one glycan. Our results show that TMEM95 is expressed and *N*-linked glycosylated in human sperm.

Using a similar approach for IZUMO1 (*SI Appendix*, Fig. S5 *A*–*C*), we generated hybridoma cell lines that produce IZUMO1-specific monoclonal antibodies, 4E04 and 6F02 (Fig. 4*D* and *SI Appendix*, Table S2). These antibodies both bind IZUMO1 (*SI Appendix*, Fig. S5 *F* and *J*) via two noncompeting epitopes (Fig. 4*E* and *SI Appendix*, Fig. S5 *D* and *E*). Compared to 4E04-bound IZUMO1-Fc, 6F02-bound IZUMO1-Fc blocks binding of IZUMO1-Fc to eggs (*SI Appendix*, Fig. S5*G*) and JUNO (*SI Appendix*, Fig. S5 *H* and *I*). These results suggest that 4E04 and 6F02 bind to different epitopes of IZUMO1, and that the 6F02 epitope overlaps with the IZUMO1-binding site for JUNO.

### TMEM95 Antibodies Impair Fusion of Human Sperm to Hamster Eggs.

**Fig. 5.**
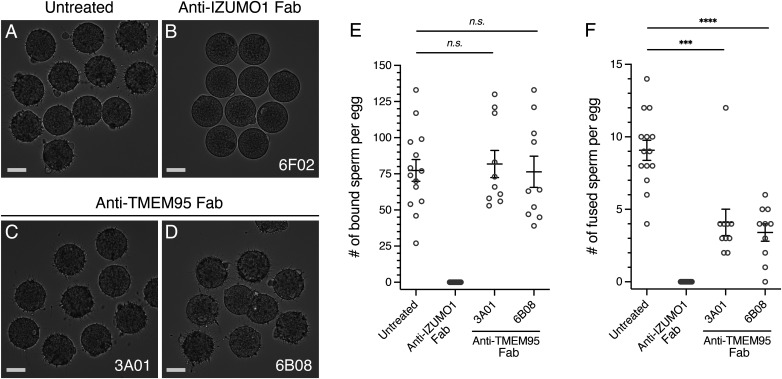
TMEM95 antibodies impair sperm-egg fusion. (*A*–*D*) Representative images showing binding of human sperm to zona-free hamster eggs (*A*) untreated or treated with 40 μg/mL of (*B*) anti-IZUMO1 Fab 6F02, (*C*) anti-TMEM95 Fab 3A01, or (*D*) anti-TMEM95 Fab 6B08. (*E*) Summary of the numbers of bound human sperm per zona-free hamster eggs (mean ± SEM), untreated 77.4 ± 7.5 (*n* = 14), anti-IZUMO1 6F02 Fab 0 ± 0 (*n* = 10), anti-TMEM95 3A01 Fab 81.8 ± 9.4 (*n* = 10, *n.s.*, not significant), and anti-TMEM95 6B08 Fab 76.4 ± 10.8 (*n* = 10, *n.s.*, not significant). (*F*) Summary of the numbers of fused human sperm per zona-free hamster eggs (mean ± SEM), untreated 9.1 ± 0.7 (*n* = 14), anti-IZUMO1 6F02 Fab 0 ± 0 (*n* = 10), anti-TMEM95 3A01 Fab 4.1 ± 0.9 (*n* = 10, *P* = 0.0002), and anti-TMEM95 6B08 Fab 3.4 ± 0.6 (*n* = 10, *P* < 0.0001). TMEM95 antibodies do not block sperm-egg binding but impair sperm-egg fusion (*SI Appendix*, Fig. S6).

**Fig. 6.**
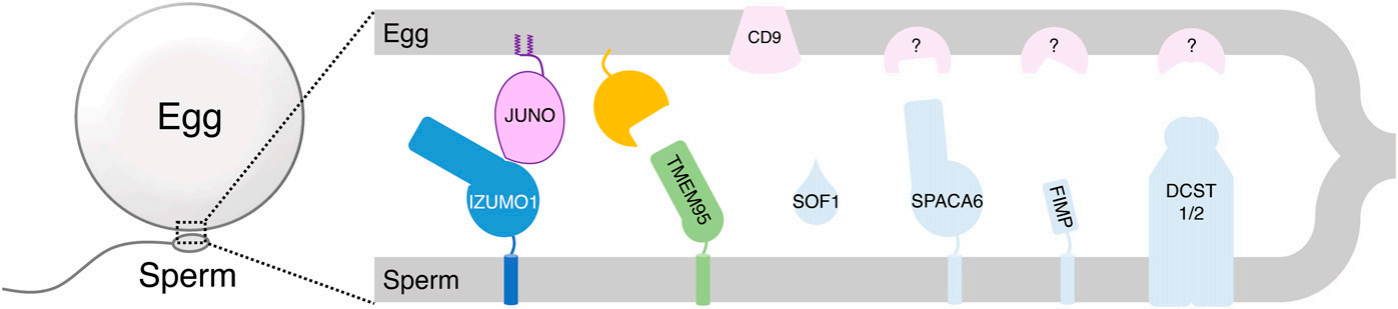
Model of sperm-egg binding and fusion. Illustration of membrane fusion of sperm and an egg and pairwise protein-protein interactions: sperm IZUMO1 (blue) binds egg JUNO (pink) and a receptor (orange)-mediated interaction of sperm TMEM95 (green) to the egg takes place; membrane fusion occurs thereafter. CD9 of egg is essential for sperm-egg fusion (31–33). Additional analogous, yet to be identified, interactions between sperm proteins (e.g., SOF1, SPACA6, FIMP, and DCST1/2) and their specific egg receptors may play a role in sperm-egg binding and fusion.

To examine whether human TMEM95 plays a role in membrane fusion, we produced the fragments antigen-binding (Fab) of the TMEM95 and IZUMO1 antibodies and tested these in a sperm penetration assay. These Fab fragments bind antigens at nanomolar affinities (*SI Appendix*, Figs. S4*E* and S5*E*) and may have less steric effects in membrane fusion than their larger IgG counterparts. We inseminated hamster eggs with human sperm preincubated with the TMEM95 antibody Fab, 3A01 (Fig. 5*C*) or 6B08 (Fig. 5*D*). We used an untreated group as a negative control (Fig. 5*A*) and IZUMO1 antibody Fab 6F02-treatment as a positive control (Fig. 5*B*). Based on the numbers of bound (Fig. 5*E*) and fused (Fig. 5*F*) sperm per egg, we found that the TMEM95 antibody Fab fragments do not block binding of sperm to eggs (Fig. 5*E*).

However, the averaged numbers of fused sperm per egg significantly decreased from 9.1 ± 0.7 (mean ± SEM) in the untreated group to 4.1 ± 0.9 (*P* = 0.0002) and 3.4 ± 0.6 (*P* < 0.0001) in the TMEM95 Fab 3A01 and 6B08 groups, respectively (Fig. 5*F* and *SI Appendix*, Fig. S6 *A*–*D*). Similarly, we observed that the TMEM95 antibody IgGs do not block sperm-egg binding (*SI Appendix*, Fig. S6 *E*–*G*), but they decrease the average numbers of fused sperm per egg when compared with a control group treated with preimmune IgG (*SI Appendix*, Fig. S6 *H*–*L*). Therefore, the two noncompeting TMEM95 monoclonal antibodies do not block sperm-egg binding but impair sperm-egg fusion, suggesting that TMEM95 plays a role in sperm-egg membrane fusion.

## Discussion

### Evidence for a Receptor for TMEM95 on Eggs.

Our results provide strong evidence for the existence of a membrane-bound receptor for sperm TMEM95 on eggs. Although the receptor has yet to be identified, our structural and site-directed mutagenesis studies identify a putative receptor-binding site on TMEM95. This region has a solvent-accessible surface area of ∼1,200 Å^2^, comparable to protein surfaces that mediate many protein-protein interactions (18, 19). We envision that the TMEM95 receptor is a membrane protein with a negatively charged region on its ectodomain surface. Nevertheless, we cannot rule out potential nonprotein receptor candidates with electrostatic negative properties on the egg surface, such as phospholipids and glycans.

The bivalent TMEM95-Fc protein introduced here may be a useful reagent to facilitate the identification of the egg receptor of TMEM95. As cell-surface interactions between membrane-bound proteins are often transient and dynamic (2, 20), the avidity of a bivalent protein could serve to stabilize the potentially weak interaction of TMEM95 with its receptor. TMEM95-Fc could therefore be used as a bait for the egg receptor, for example, for coimmunoprecipitation of mammalian eggs [e.g., see (21)], or for screening cultured cells expressing an egg cDNA library [e.g., see (2)].

### Potential Roles of TMEM95 in Membrane Fusion.

The TMEM95 antibodies used in this study do not ablate binding of TMEM95 to hamster eggs. How would the nonblocking antibodies of TMEM95 inhibit sperm-egg fusion? One possibility is that TMEM95 undergoes structural changes that are important for membrane fusion. Should sperm-egg fusion be accompanied by changes of TMEM95 in protein conformation or oligomeric state, the antibodies raised here against a defined conformation of TMEM95 may trap TMEM95 in a prefusion, monomeric state. Notably, early studies have suggested essential structural changes for IZUMO1 (e.g., rearrangement of disulfides, protein dimerization) during sperm-egg membrane fusion (14, 22, 23).

Alternatively, or in addition, TMEM95 may assemble into a complex with other sperm proteins, such as a membrane fusogen. Antibody binding to TMEM95 could affect these events and explain the inhibitory results. Additionally, these antibodies might create steric hinderance which could interfere with membrane fusion (note, however, that an anti-IZUMO1 IgG, Mab125, does not block sperm-egg fusion, (24)).

Taken together, we conceptualize that sperm-egg membrane fusion involves pairwise cell surface interactions. Sperm IZUMO1 binds egg JUNO, which mediates gamete adhesion, and a receptor-mediated interaction of sperm TMEM95 to the egg takes place; membrane fusion occurs thereafter. We anticipate additional analogous, yet to be identified, interactions between sperm proteins (6, 25–28) and their specific egg receptors (Fig. 6).

In summary, our results suggest that human sperm TMEM95 likely plays a direct role in membrane fusion with eggs. Future work is needed to rule out indirect effects of TMEM95 antibodies that inhibit fusion while not blocking sperm-egg binding. More broadly, our work takes steps toward fully understanding the molecular interactions of the fertilization complex and has implications for the development of infertility treatments and contraceptives.

## Materials and Methods

Additional information is provided in *SI Appendix*, *Materials and Methods*.

### Immunofluorescence Microscopy of Hamster Eggs.

Sexually mature female Syrian golden hamsters (Japan SLC Inc.) (approved by the Animal Care and Use Committee of the Research Institute for Microbial Diseases, Osaka University #28-4-2) were superovulated by peritoneal injection of pregnant mare serum gonadotropin and human coagulating gland (20 units for each; ASKA Pharmaceutical). Cumulus-oocyte complexes were extracted from the oviductal ampulla and treated with 1 mg/mL collagenase to remove the cumulus cells and zona pellucida, which yields zona-free eggs. These zona-free eggs were incubated with 200 nM Fc-fusion proteins in Biggers-Whitten-Whittingham medium (11) for 1 h and then stained with goat anti-human IgG Fc antibody DyLight 488 (Invitrogen) at a dilution of 1:50 for 1 h at 37 °C, 5% CO_2_. The eggs were imaged under a Keyence BZ-X810 microscope.

### Protein Crystallization of TMEM95.

Native TMEM95 proteins were crystallized at room temperature in a sitting-drop vapor diffusion system. Three hundred fifty nanoliters of 6.8 mg/mL protein was mixed with 350 nL of a reservoir solution of 150 mM NaCl, 20 mM Hepes pH 7.3, 30 mM CaCl_2_, 2% (wt/vol) PPG-P400, and 22% (wt/vol) PEG 3,350, over 80 μL of reservoir solution. Native crystals were supplemented with 20% (wt/vol) PEG 400 before cryo-cooling in liquid nitrogen. For multiwavelength anomalous diffraction, crystals were grown in 150 mM NaCl, 20 mM Hepes pH 7.3, 10 mM CaCl_2_, 2% (wt/vol) PPG-P400, and 18% (wt/vol) PEG 3,350, and were transferred to a solution supplemented with 500 mM SmCl_3_ and incubated for ∼5 min. The Sm^3+^-bound crystals were washed in a SmCl_3_-free reservoir solution, cryo-protected with 20% PEG 400, and cooled in liquid nitrogen.

### Sperm Penetration Assay.

Sperm penetration assays were performed as previously described (11) with minor changes. Briefly, human semen from healthy donors, who had informed consent, was liquefied for 30 min at room temperature. The sperm were purified by discontinuous Percoll gradients (29) and incubated in Biggers-Whitten-Whittingham medium containing 2.5 μM calcium ionophore for 3 h at 37 °C, 5% CO_2_. The sperm were washed in fresh Biggers-Whitten-Whittingham medium and treated with monoclonal antibodies at 40 μg/mL for 1 h at 37 °C, 5% CO_2_. Motile sperm were manually counted in a hemocytometer under an inverted microscope. Zona-free hamster eggs were inseminated in 100 μL medium drops by the antibody-treated sperm at a density of 3 × 10^6^ motile sperm per milliliter for 3 h at 37 °C, 5% CO_2_. The eggs were washed in fresh medium, gently flattened by coverslips, and examined under a phase-contrast microscope.

## Supplementary Material

Supplementary File

## Data Availability

The coordinate and structure factor of human sperm TMEM95 ectodomain has been deposited in the RCSB Protein Data Bank under PDB ID code 7UX0 (34). The structure is available immediately at https://peterkimlab.stanford.edu (35).
