# Supplementary material for: Human sperm TMEM95 binds eggs and facilitates membrane fusion
Source: Proc Natl Acad Sci U S A. 2022 Sep 26;119(40):e2207805119. doi: 10.1073/pnas.2207805119 (PMC9546558; doi:10.1073/pnas.2207805119)
Supplement: Supplementary File [file pnas.2207805119.sapp.pdf]

PNAS

Supplementary Information for

Human sperm TMEM95 binds eggs and facilitates membrane fusion

Shaogeng Tang, Yonggang Lu, Will M. Skinner, Mrinmoy Sanyal,

Polina V. Lishko, Masahito Ikawa, Peter S. Kim

Peter S. Kim, Email: kimpeter@stanford.edu

Masahito Ikawa, Email: ikawa@biken.osaka-u.ac.jp

This PDF file includes:

SI Materials and Methods

Fig. S1, related to Fig. 1, Characterization of TMEM95-Fc and IZUMO1-Fc

Fig. S2, related to Fig. 2, JUNO does not act as a receptor of TMEM95

Fig. S3, related to Fig. 3, Characterization of TMEM95-Fc variants

Fig. S4, related to Fig. 4, Characterization of the TMEM95 antibodies

Fig. S5, related to Fig. 4, Characterization of the IZUMO1 antibodies

Fig. S6, related to Fig. 5, TMEM95 antibodies impair sperm-egg fusion

Table S1 Crystallographic data collection and refinement statistics

Table S2 Summary of the TMEM95 and IZUMO1 monoclonal antibodies

Table S3 Plasmids and protein sequences used in this study

SI References

SI Materials and Methods

Expression and purification of the Fc-fusion proteins

The cDNAs encoding human TMEM95 (residues 1-145) or human IZUMO1 (residues 1-255) were subcloned into a pADD2 vector that carries a C-terminal fusion of a TEV protease cleavage site, a human IgG1 Fc, an Avi tag, and a hexa-histidine tag (*SI Appendix*, Table S3). The recombinant Fc-fusion proteins were overexpressed by transient transfection of HEK293F cells (ThermoFisher) cultured at 37 °C, 8% CO_2. TMEM95-Fc was purified by Ni-NTA affinity purification (Invitrogen), followed by anion exchange using an AKTA pure system by a Mono Q 5/50 GL (Cytiva). IZUMO1-Fc was purified by Protein-A affinity purification using a MabSelect Prism (Cytiva), followed by anion exchange using a Mono Q 5/50 GL. Purified proteins were stored in a buffer of 150 mM NaCl, 20 mM HEPES pH 7.4.

Tagless IZUMO1 proteins were obtained from IZUMO1-Fc through TEV (Sigma-Aldrich) cleavage overnight at 4 °C. Undigested proteins and the histidine tagged TEV proteases were removed by a MabSelect Prism followed by a HisTrap excel (Cytiva). Tagless IZUMO1 was further purified by gel filtration with a Superdex 200 Increase 10/300 GL (Cytiva) in a buffer of 150 mM NaCl, 20 mM HEPES pH 7.4.

Immunofluorescence microscopy of murine eggs

Sexually mature female B6D2F1 mice (Japan SLC Inc.) (approved by the Animal Care and Use Committee of the Research Institute for Microbial Diseases, Osaka University #Biken-AP-H30-01) were superovulated by peritoneal injection of pregnant mare serum gonadotropin and human coagulating gland (20 units for each; ASKA Pharmaceutical).

Cumulus-oocyte complexes were extracted from the oviductal ampulla and treated with 1 mg/mL collagenase to remove the cumulus cells and zona pellucida, which yields zona-free eggs. These zona-free eggs were incubated with 200 nM TMEM95 in Toyoda-Yokoyama-Hoshi medium (1) for 1 h and then stained with goat anti-human IgG Fc antibody DyLight 488 (Invitrogen) at a dilution of 1:50 for 1 h at 37 °C, 5% CO_2. The eggs were imaged under a Keyence BZ-X810 microscope.

Protein expression and purification of JUNO

The cDNA encoding human JUNO (residues 20-227) was subcloned into a baculoviral vector pACgp67a that carries a signal sequence of MVSAIVLYVLLAAAAHSAFA and C-terminal hexa-histidine tag (*SI Appendix*, Table S3). Baculovirus was generated from Sf9 cells (ThermoFisher) by a co-transfection of pACgp67a and the BestBac Linearized Baculovirus DNA (Expression Systems). Passage one baculovirus was tittered and used for infecting HighFive cells (ThermoFisher) cultured at 27 °C. ~3 days post infection, the conditioned media were harvested and mixed with $NiCl_2$, $CaCl_2$, and Tris pH 8.0 to a final concentration of 1 mM, 5 mM, and 100 mM, respectively. After centrifugation, the JUNO-His_6 proteins was purified by Ni-NTA affinity purification from the resulting supernatant, followed by gel filtration with a Superdex 200 Increase 10/300 GL in a buffer of 150 mM NaCl, 20 mM HEPES pH 7.4.

Biolayer interferometry

An Octet RED96 system (Pall ForteBio) was employed for protein-protein interaction assays in a buffer of 150 mM NaCl, 20 mM HEPES pH 7.4, 0.1% bovine serum

albumin, and 0.05% Tween 20 at 29 °C under a shaking speed of 1,000 rpm. Biotinylated TMEM95-Fc or IZUMO1-Fc proteins were loaded onto Streptavidin biosensors (Sartorius). After loading the biosensors were baselined, associated in defined concentrations of analytes, and dissociated in the buffer with no analytes. Baseline-corrected binding traces were plotted and analyzed using GraphPad Prism 9.

Differential scanning fluorimetry

A Prometheus NT.48 (NanoTemper) was employed for nanoscale differential scanning fluorimetry (NanoDSF). Protein samples were loaded into capillaries and subject to a temperature from 20 to 95 °C at a heating rate of 1 °C/min. Intrinsic fluorescence at 350 nm and 330 nm was recorded as a function of temperature. Thermal melting profiles were plotted using the first derivative of the ratio ($F_{350\ \mathrm{nm}}/F_{330\ \mathrm{nm}}$). Melting temperatures were calculated by the instrument and represented peaks in the thermal melting curves.

Protein purification of TMEM95

The cDNA encoding human TMEM95 (residues 17-138) was subcloned into a pADD2 vector. The N-terminus of TMEM95 was fused to a signal sequence of MRMQLLLLIALSLALVTNS and the C-terminus to a C-tag of EPEA (*SI Appendix*, Table S3). Recombinant TMEM95 proteins were overexpressed in HEK293F cells by transient transfection. Affinity purification was performed using the CaptureSelect C-tagXL affinity matrix (ThermoFisher). The eluate was purified by cation exchange by a Mono S 5/50 GL (Cytiva), followed by gel filtration with a Superdex 200 Increase 10/300 GL in a buffer of 150 mM NaCl, 20 mM HEPES pH 7.4. Size exclusion with multiangle light

scattering was performed on an Agilent 1260 Infinity II high performance liquid chromatography coupled with Wyatt detectors for light scattering (miniDAWN) and refractive index (Optilab) measurements.

X-ray crystallography

X-ray diffraction data were collected at the Stanford Synchrotron Radiation Lightsource (SSRL) beam line 12-2 of SLAC National Accelerator Laboratory. For the Sm^{3+}-bound crystal, multi-wavelength anomalous diffraction data were collected at wavelengths 1.694 Å (peak), 1.137 Å (remote), and 1.695 Å (inflection). For the native crystal, the diffraction data were collected at 0.979 Å wavelength to 1.50 Å resolution. All diffraction data were processed using *autoPROC* (2). The TMEM95 ectodomain structure was solved by experimental phasing using *AutoSol* in *Phenix* (3). An initial model containing 110 amino acids and two Sm^{3+} ions were obtained using *AutoBuild* and was subsequently applied to the native X-ray dataset by molecular replacement using *Phaser*. Model refinement and density modification were performed in *Phenix*. Model building was performed using *Coot* (4). Structural illustrations were generated with *PyMOL*.

Evolutionary conservation by *CONSURF*

The protein sequence of human TMEM95 was input as a query sequence for a protein *BLAST* search using blastp. The top 150 results from were filtered manually and ortholog-unique sequences were subjected for alignment by *MAFFT*. The multiple sequence alignment and the TMEM95 structure were used as input in the *CONSURF*

server (5). The overall conservation scores from 1 to 9 were calculated using the Bayesian methods for each amino acid and were mapped onto the TMEM95 structure in a color-coordinated fashion as shown in Fig. 3.

Generation of mouse hybridomas

Five female BALB/c mice (Jackson Laboratory) aged ~8 weeks (approved by Stanford University Administrative Panel on Laboratory Animal Care, APLAC 33984) were immunized with 10 μg purified protein of TMEM95 (residues 17-138) in 100 μL of 150 mM NaCl, 20 mM HEPES pH 7.4, adjuvanted with 10 μg Quil-A (InvivoGen) and 10 μg monophosphoryl lipid A (InvivoGen). Mice were boosted at days 21, 43, 64, and 86. At day 90, a spleen of one mouse was disaggregated into a single-cell suspension for hybridoma generation following the manufacturer's procedures (Stemcell technologies). Briefly, splenocytes were purified and fused with Sp2/0-Ag14 cells (ATCC) using polyethylene glycol. Hybridomas were cultured in 96-well plates with a selection medium containing hypoxanthine, aminopterin, and thymidine. ~14 days after recovery, the conditioned media were screened for binding to TMEM95 by ELISA. TMEM95-binding-positive cells were sorted as single cells in 96-well plates using a SONY SH800S. ~14 days after recovery, the conditioned media were screened, and the selected TMEM95-positive clones were expanded for antibody sequencing (Genscript Biotech) (*SI Appendix*, Table S2). Similarly, five mice were immunized with IZUMO1 (residues 22-255) and boosted at days 21, 43, 61, and 96. At day 100, a spleen from one mouse was used for hybridoma generation.

Antibody production and purification

Hybridomas producing the TMEM95 and IZUMO1 antibodies were cultured in ClonaCell-HY Medium E (Stemcell technologies) and subsequently adapted to serum-free AOF Expansion Medium (Stemcell technologies) for 5-7 days at 37 °C, 5% CO_2. The IgG in the conditioned AOF media was harvested from the supernatants and subjected for affinity purification by a HiTrap Protein G HP (Cytiva) and gel filtration with a Superdex 200 Increase 10/300 GL in a buffer of 150 mM NaCl, 20 mM HEPES pH 7.4.

The cDNAs encoding the heavy and light chains of the TMEM95 and IZUMO1 antibody Fab were subcloned into a pVRC vector (*SI Appendix*, Table S3). The Fabs were produced in HEK293F cells by transient transfection at 37 °C, 8% CO_2, and purified from the supernatants of the conditioned media by a HiTrap Protein G HP, followed by gel filtration with a Superdex 200 Increase 10/300 GL in a buffer of 150 mM NaCl, 20 mM HEPES pH 7.4. All antibodies were concentrated to 1.0 mg/mL, supplemented with 10% glycerol, and aliquoted for long-term storage at -80 °C.

Human sperm isolation and western blotting

The experimental procedures utilizing human derived samples in Fig. 4 and *SI Appendix* Figs. S4 and S5 were approved by the Committee on Human Research at the University of California, Berkeley, IRB protocol 2013-06-5395. Purified human sperm (6) were lysed in a buffer of 150 mM NaCl, 50 mM Tris pH 7.4, 1% Triton X-100, 0.5% Sodium deoxylcholate, 0.1% SDS, 1 mM EDTA, 10% (v/v) glycerol, and Halt protease inhibitors (ThermoFisher). The protein concentrations of the whole cell lysates were

estimated by a Bradford assay (BioRad) using bovine serum albumin as a standard. The lysates were stored at 4 °C in a non-reducing condition before loaded onto an SDS-PAGE gel for electrophoresis. 15 µg of lysates and 10 µg/mL of TMEM95 antibodies were used for the detection of TMEM95; 7 µg of lysates and 2 µg/mL of IZUMO1 antibodies were used for the detection of IZUMO1. A secondary antibody of HRP-conjugated goat anti-mouse IgG (BioLegend) was used for immunoblotting. PNGaseF treatment was performed under non-reducing conditions following the manufacturer's instructions (NEB).

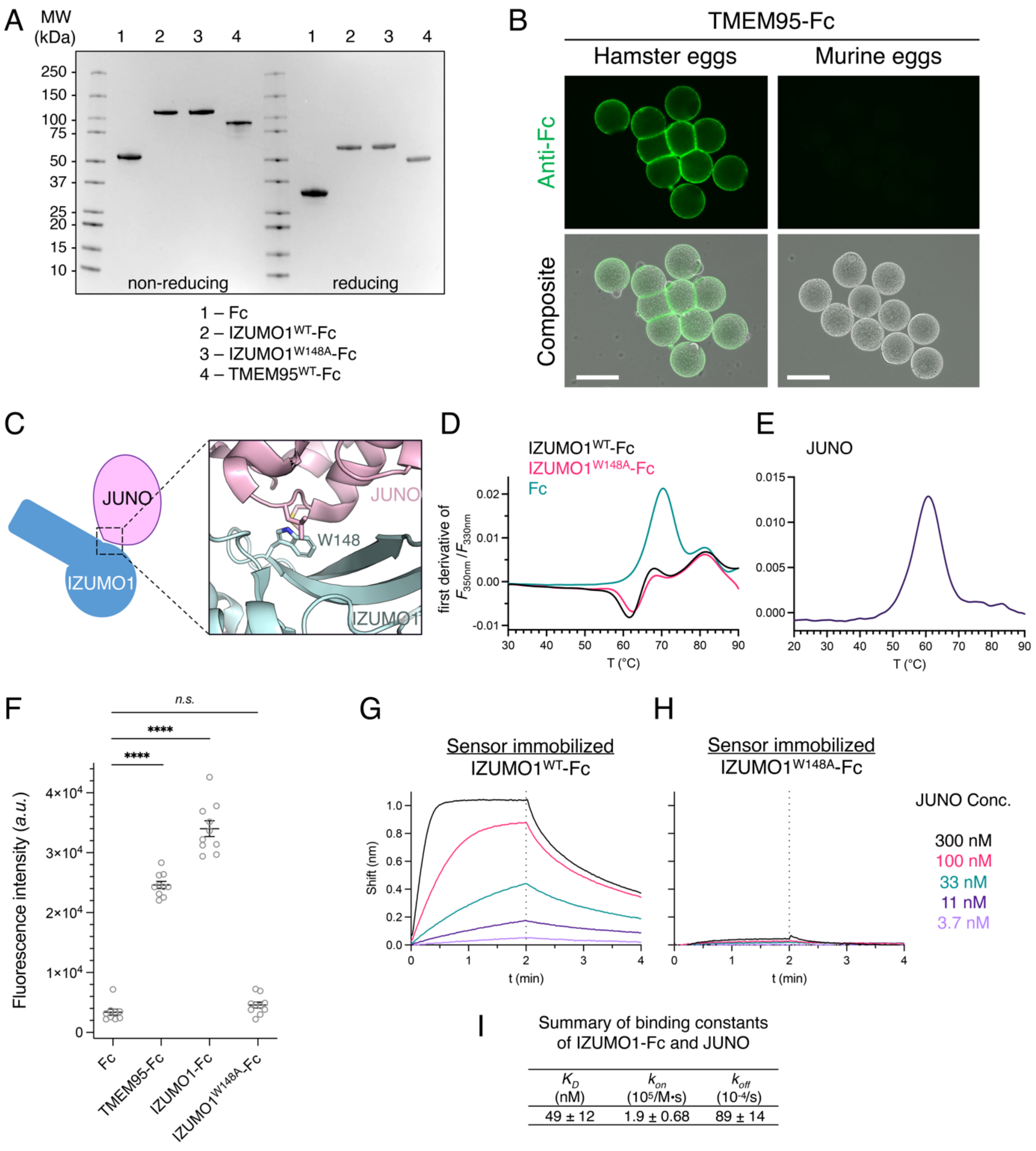

Fig. S1, related to Fig. 1, Characterization of TMEM95-Fc and IZUMO1-Fc. (A) Coomassie-blue stained SDS-PAGE gel of Fc, IZUMO1^{WT}-Fc, IZUMO1^{W148A}-Fc, and TMEM95-Fc proteins under non-reducing (left) or reducing conditions (right). (B) Immuno-fluorescence (upper) and differential interference contrast composite images

(lower) of (left) zona-free hamster eggs and (right) zona-free mouse eggs were incubated with 200 nM TMEM95-Fc. Green fluorescence by a DyLight 488-conjugated anti-Fc antibody. Scale bars, 100 μm. (C) Cartoon schematic and ribbon diagram (PDB ID: 5F4E) of the IZUMO1-JUNO complex showing the side chain of W148 of IZUMO1 interacting with JUNO. (D-E) NanoDSF thermal melting profiles of (D) Fc, $IZUMO1^{WT}$-Fc, $IZUMO1^{W148A}$-Fc, and (E) JUNO proteins. (F) Quantification of fluorescence intensities (*a.u.*, arbitrary unit; ****, $p < 0.0001$; *n.s.*, not significant) of Fc, TMEM95-Fc, $IZUMO1^{WT}$-Fc, and $IZUMO1^{W148A}$-Fc on eggs shown in Fig. 1. (G-H) Biolayer interferometric traces of sensor immobilized (G) $IZUMO1^{WT}$-Fc or (H) $IZUMO1^{W148A}$-Fc binding JUNO of 300 nM, 100 nM, 33 nM, 11 nM, and 3.7 nM, with association for 2 min and dissociation for 2 min. WT, wild type. (I) List of binding constants of IZUMO1-Fc with JUNO calculated from traces in (G).

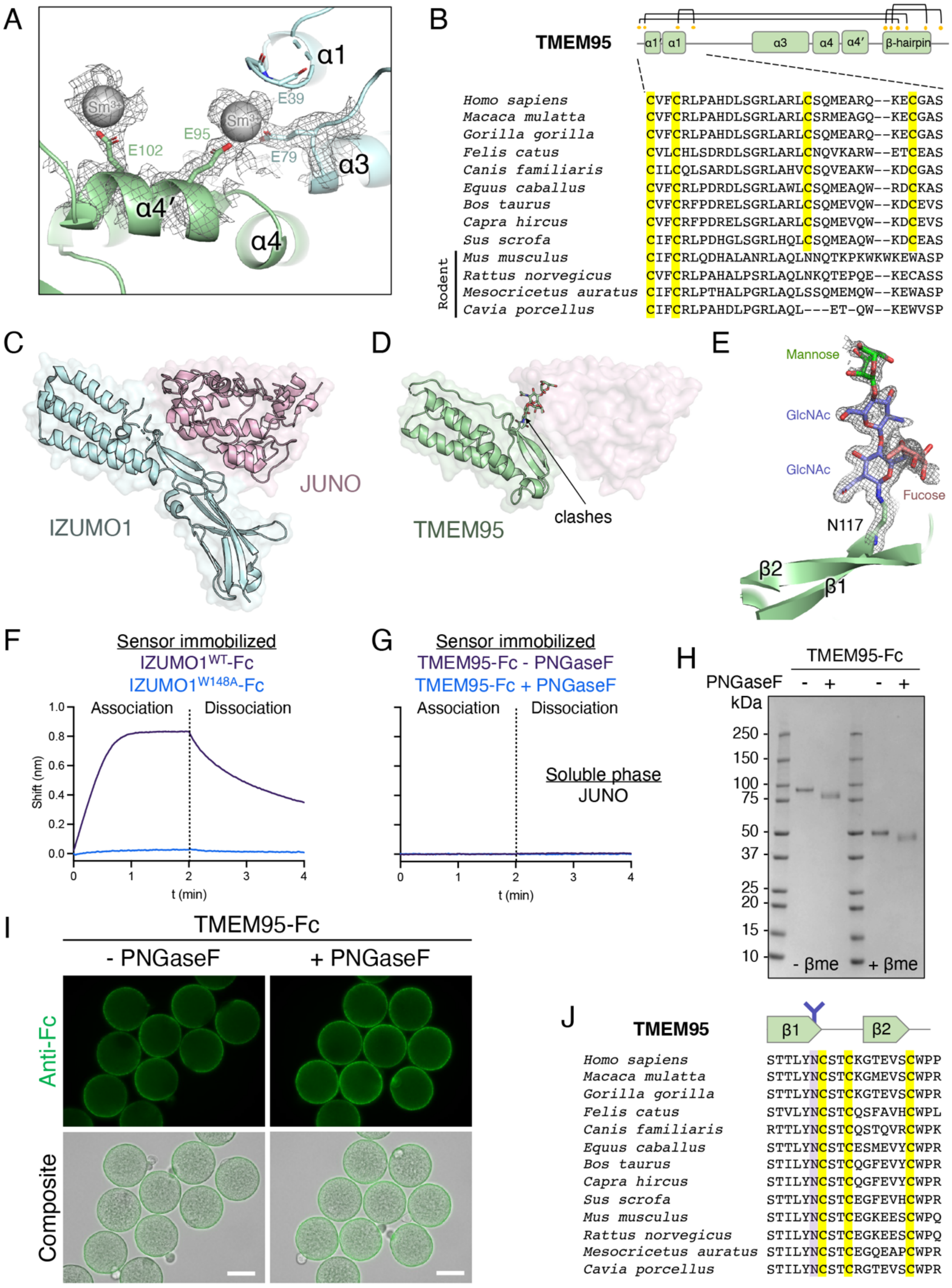

Fig. S2, related to Fig. 2, JUNO does not act as a receptor of TMEM95. (A) Ribbon diagram overlay with a $2F_{obs}$-F_{calc} electron density map surrounding the Sm^{3+} ions

between two TMEM95 protomers (green and cyan) in the crystal lattice solved by multi-wavelength X-ray anomalous diffraction. (B) Multiple sequence alignment of the α1 region of TMEM95 orthologs with conserved cysteines highlighted in yellow. (C-D) Ribbon diagrams overlay with a space-filling model of (C) the IZUMO1-JUNO complex (PDB ID: 5F4E), (D) the TMEM95-superimposed JUNO complex, where the N-glycan of TMEM95 causes a clash with JUNO. (E) Ribbon diagram overlay with a $2F_{obs}$-F_{calc} composite omit (10%) electron density map of the N117 side chain and its linked glycan. Biolayer interferometric traces of (F) sensor immobilized IZUMO1^{WT}-Fc or IZUMO1^{W148A}-Fc, and (G) sensor immobilized TMEM95-Fc treated without or with PNGaseF binding to JUNO of 300 nM, with association for 2 min and dissociation for 2 min. Data in (F) are also shown in *SI Appendix*, Fig. S1G-H. (H) Coomassie-blue stained SDS-PAGE gel of TMEM95-Fc treated without or with PNGaseF under non-reducing (left) or reducing (right) conditions. (I) Immuno-fluorescence (upper) and differential interference contrast composite images (lower) of zona-free hamster eggs incubated with TMEM95-Fc treated without PNGaseF (left) and with PNGaseF (right). Green fluorescence by a DyLight 488-conjugated anti-Fc antibody. Scale bars, 50 μm. (J) Multiple sequence alignment of the β-hairpin of TMEM95 orthologs with conserved cysteines highlighted in yellow and the asparagine in purple.

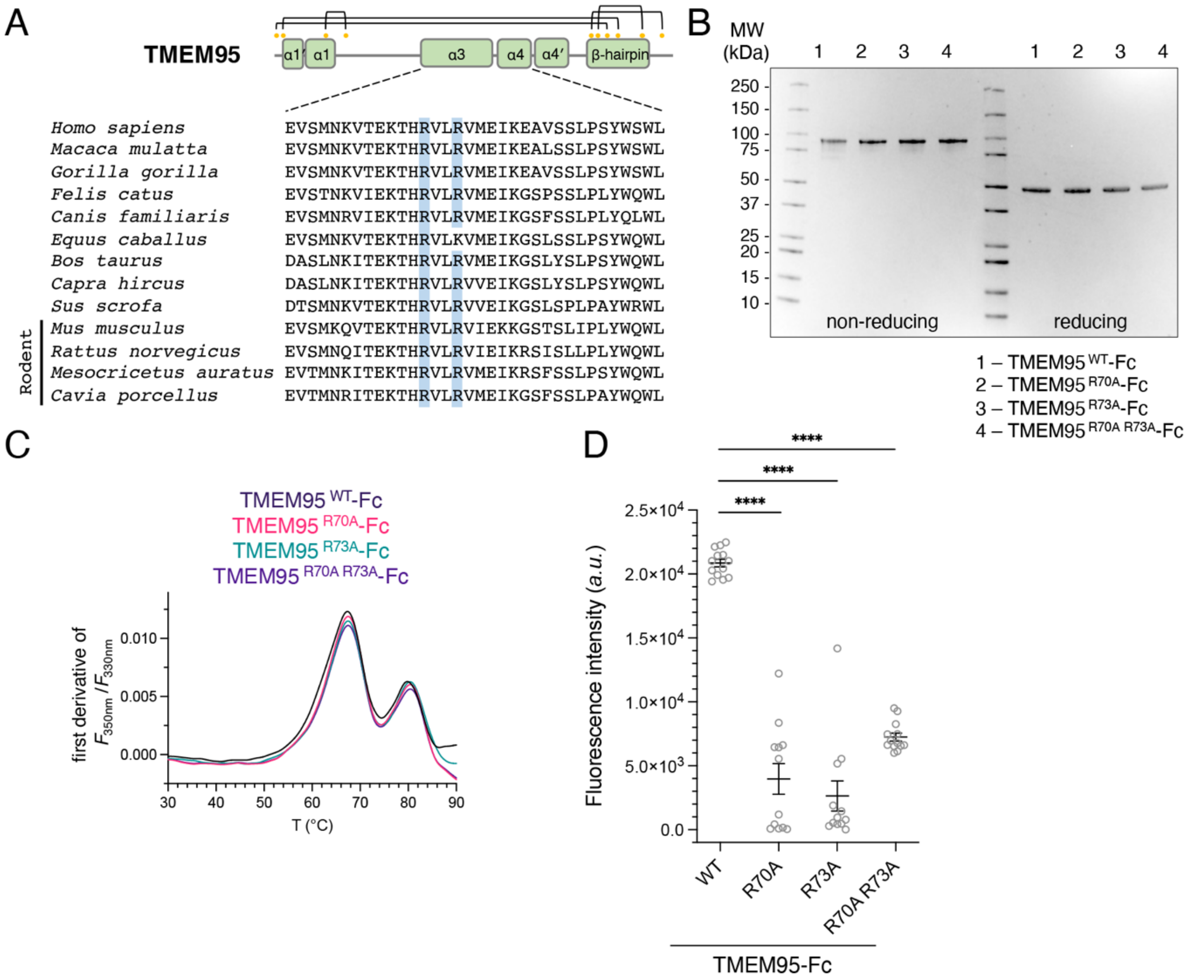

Fig. S3, related to Fig. 3, Characterization of TMEM95-Fc variants. (A) Multiple sequence alignment of the α3-α4 region of TMEM95 orthologs with conserved arginine highlighted in blue. (B) Coomassie-blue stained SDS-PAGE gel of TMEM95^{WT}-Fc, TMEM95^{R70A}-Fc, TMEM95^{R73A}-Fc, and TMEM95$^{R70A\ R73A}$-Fc proteins under non-reducing (left) or reducing (right) conditions. WT, wild type. (C) NanoDSF thermal melting profiles of TMEM95^{WT}-Fc, TMEM95^{R70A}-Fc, TMEM95^{R73A}-Fc, and TMEM95$^{R70A\ R73A}$-Fc proteins. (D) Quantified green fluorescence intensities (*a.u.*, arbitrary unit; ****, $p < 0.0001$) of TMEM95^{WT}-Fc, TMEM95^{R70A}-Fc, TMEM95^{R73A}-Fc, and TMEM95$^{R70A\ R73A}$-Fc proteins on eggs shown in Fig. 3.

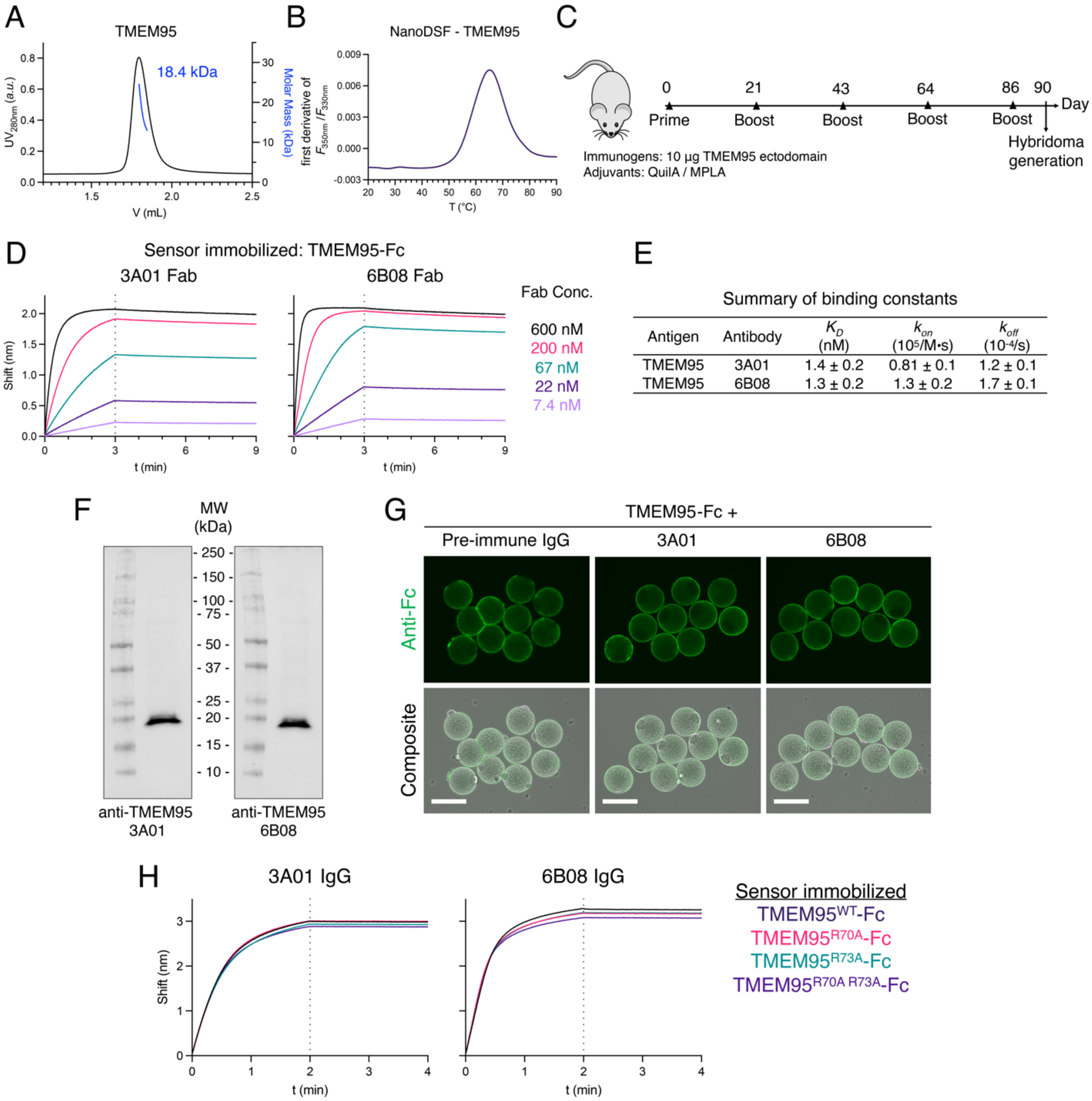

Fig. S4, related to Fig. 4, Characterization of the TMEM95 antibodies. (A) Size exclusion and multiangle light scattering of the TMEM95 protein showing a monodispersed peak with a calculated molecular weight of 18.4 kDa, as an expected monomer in solution. (B) NanoDSF thermal melting profile of the TMEM95 protein used for protein crystallization and mouse immunization. (C) Schedule of mouse immunization using the TMEM95 protein. (D) Biolayer interferometric traces of sensor

immobilized TMEM95-Fc binding to 3A01 Fab or 6B08 Fab at concentrations of 600 nM, 200 nM, 67 nM, 22 nM, and 7.4 nM, with association for 3 min and dissociation for 6 min. (E) Summary of binding constants of TMEM95-Fc with anti-TMEM95 3A01 Fab and 6B08 Fab calculated from traces in (D). (F) Western blots of non-heat-denatured, non-reduced human sperm lysates by a primary antibody of 10 μg/mL anti-TMEM95 3A01 IgG or 6B08 IgG, and a secondary HRP-conjugated anti-mouse antibody. (G) Immuno-fluorescence (upper) and differential interference contrast composite images (lower) of zona-free hamster eggs incubated with TMEM95-Fc that has been pre-bound to protein G purified pre-immune mouse IgG, anti-TMEM95 3A01 IgG, or 6B08 IgG. 2.5 μM TMEM95-Fc was mixed with 5 μM (0.75 mg/mL) IgG for 1 hour to form a complex of TMEM95-Fc and the antibody, and the mixture was added to the eggs at a final concentration of 200 nM TMEM95-Fc. Green fluorescence by a DyLight 488-conjugated anti-Fc antibody. Scale bars, 100 μm. (H) Biolayer interferometric traces of sensor-immobilized $TMEM95^{WT}$-Fc, $TMEM95^{R70A}$-Fc, $TMEM95^{R73A}$-Fc, and $TMEM95^{R70A\ R73A}$-Fc proteins binding to 200 nM of 3A01 IgG or 6B08 IgG, with association for 2 min and dissociation for 2 min. WT, wild type.

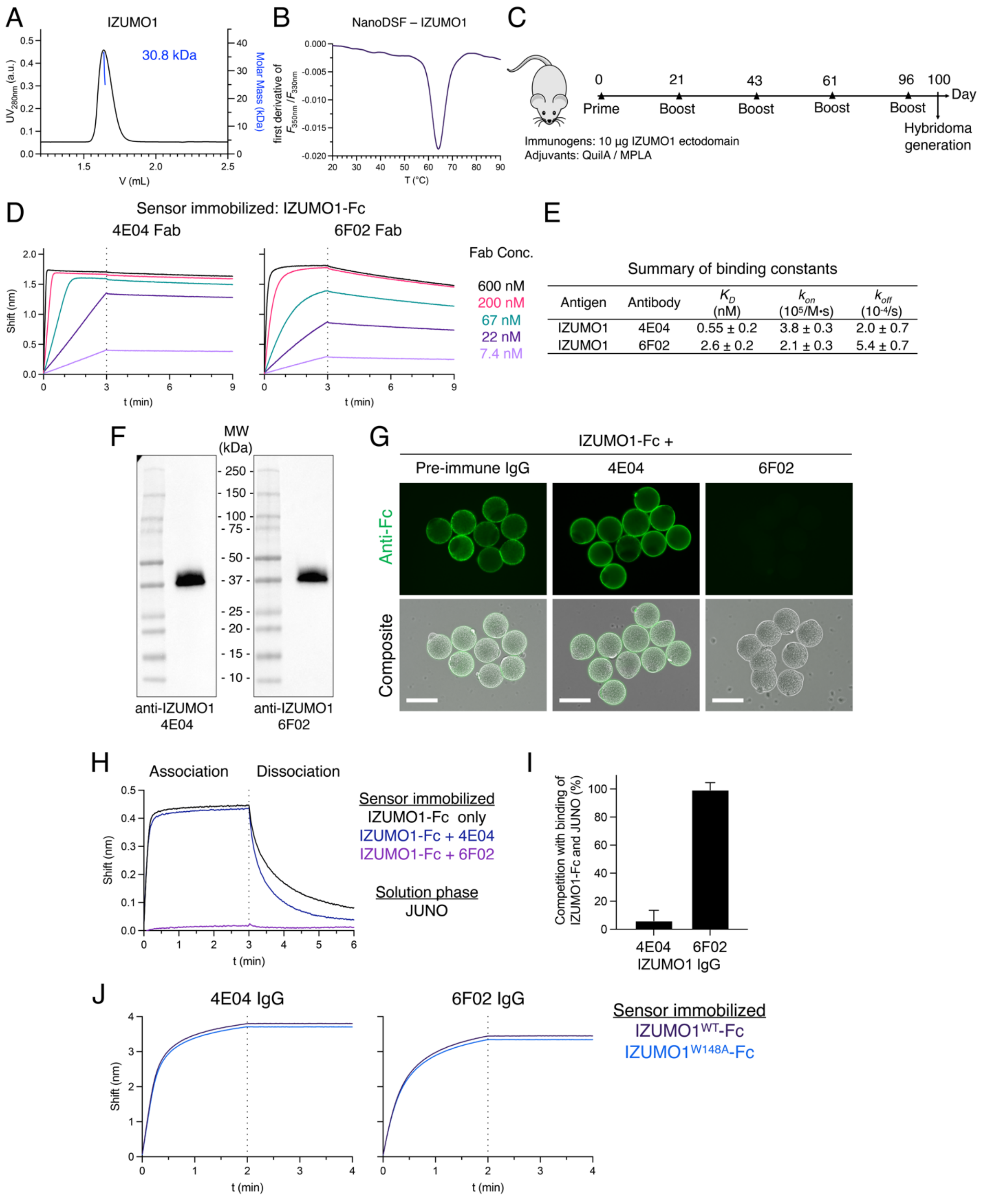

Fig. S5, related to Fig. 4, Characterization of the IZUMO1 antibodies. (A) Size exclusion and multiangle light scattering of the IZUMO1 protein showing a monodispersed peak

with a calculated molecular weight of 30.8 kDa, as an expected monomer in solution. (B) NanoDSF thermal melting profile of the IZUMO1 protein used for mouse immunization. (C) Schedule of mouse immunization of the IZUMO1 protein. (D) Biolayer interferometric traces of sensor immobilized IZUMO1-Fc binding to 4E04 Fab or 6F02 Fab at concentrations of 600 nM, 200 nM, 67 nM, 22 nM, and 7.4 nM, with association for 3 min and dissociation for 6 min. (E) Summary of binding constants of IZUMO1-Fc with anti-IZUMO1 4E04 Fab and 6F02 Fab calculated from traces in (D). (F) Western blots of non-heat-denatured, non-reduced human sperm lysates by a primary antibody of 2 μg/mL anti-IZUMO1 4E04 IgG or 6F02 IgG, and a secondary HRP-conjugated anti-mouse antibody. (G) Immuno-fluorescence (upper) and differential interference contrast composite images (lower) of zona-free hamster eggs incubated with IZUMO1-Fc that has been pre-bound to protein G purified pre-immune mouse IgG, anti-IZUMO1 4E04 IgG, or 6F02 IgG. 2.5 μM IZUMO1-Fc was mixed with 5 μM (0.75 mg/mL) IgG for 1 hour to form a complex of IZUMO1-Fc and the antibody, and the mixture was added to the eggs at a final concentration of 200 nM IZUMO1-Fc. Green fluorescence by a DyLight 488-conjugated anti-Fc antibody. Scale bars, 100 μm. (H) Biolayer interferometric traces of sensor immobilized IZUMO1-Fc, or IZUMO1-Fc in complex with 4E04 IgG, 6F02 IgG binding to 300 nM JUNO, with association for 3 min and dissociation for 3 min. (I) Summary of antibody competition with the IZUMO1-Fc and JUNO interaction calculated from (H). (J) Biolayer interferometric traces of sensor-immobilized IZUMO1^{WT}-Fc and IZUMO1^{W148A}-Fc binding to 200 nM of 4E04 IgG or 6F02 IgG, with association for 2 min and dissociation for 2 min. WT, wild type.

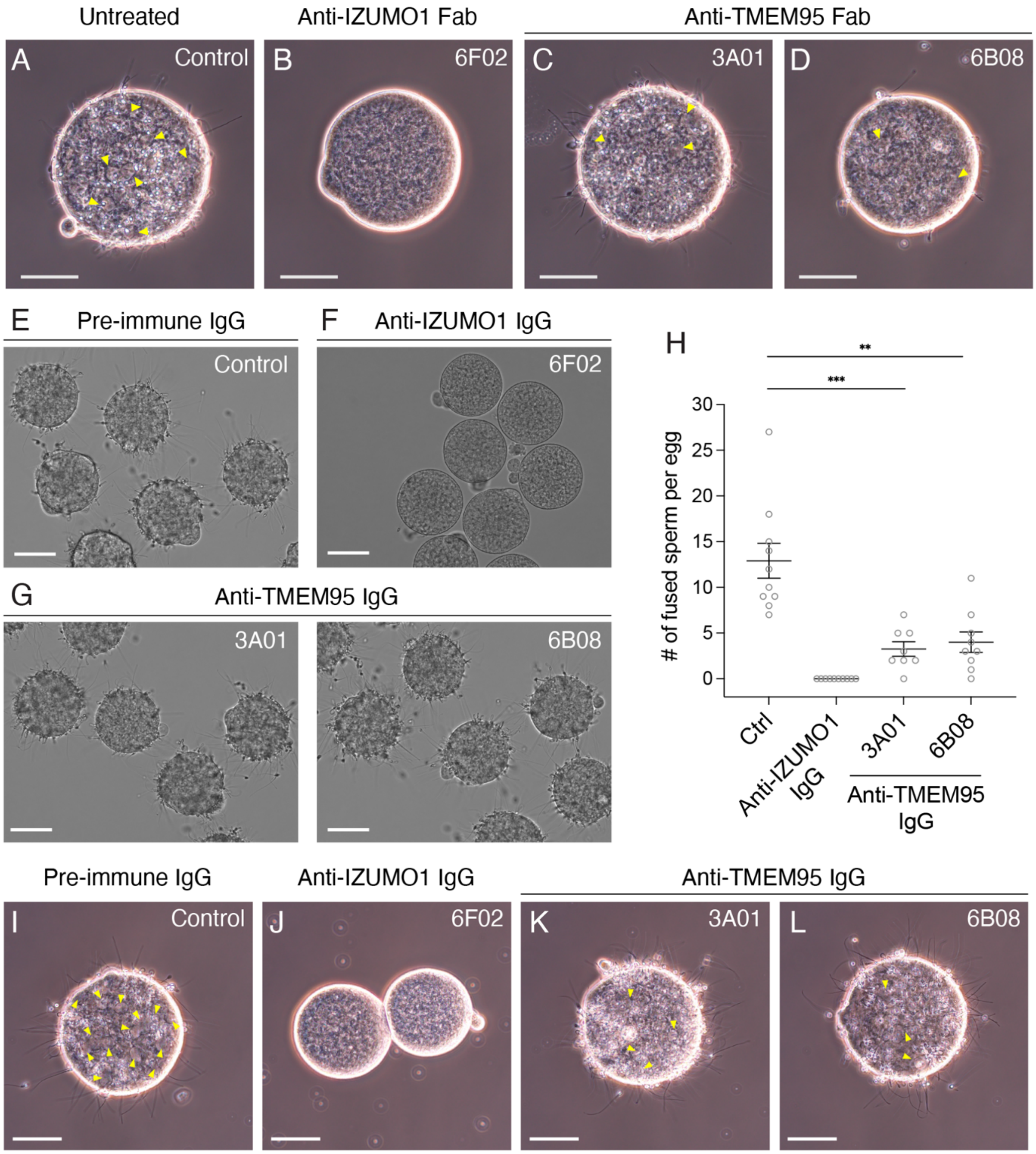

Fig. S6, related to Fig. 5, TMEM95 antibodies impair sperm-egg fusion. Representative images showing fusion of human sperm with zona-free hamster eggs (A) untreated or treated with 40 µg/mL of (B) anti-IZUMO1 Fab 6F02, (C) anti-TMEM95 Fab 3A01, or (D) anti-TMEM95 Fab 6B08. Arrows indicating fused sperm with swollen sperm heads. Scale bars, 50 µm. (E-G) Representative images showing binding of human sperm with

zona-free hamster eggs in the presence of 40 µg/mL (E) pre-immune mouse IgG, (F) anti-IZUMO1 IgG 6F02, (G) anti-TMEM95 IgG 3A01 (left), or anti-TMEM95 IgG 6B08 (right) after 3 hours of insemination. Scale bars, 50 µm. (H) Summary of the numbers of fused human sperm per zona-free hamster eggs in each group (mean ± SEM), pre-immune mouse IgG control 12.9 ± 1.7 (N = 10), anti-IZUMO1 IgG 6F02 0 ± 0 (N = 10), anti-TMEM95 IgG 3A01 3.3 ± 0.8 (N = 8, $p < 0.001$), and anti-TMEM95 IgG 6B08 4.0 ± 1.1 (N = 9, $p < 0.01$). Representative images showing fusion of human sperm with zona-free hamster eggs in the presence of 40 µg/mL (I) pre-immune mouse IgG, (J) anti-IZUMO1 IgG 6F02, (K) anti-TMEM95 IgG 3A01, and (L) anti-TMEM95 IgG 6B08. Arrows indicating fused sperm with swollen sperm heads. Scale bars, 50 µm.

Table S1 Crystallographic data collection and refinement statistics

	Human TMEM95 ectodomain			
	Native	Multi-wavelength anomalous diffraction		
		Peak	Remote	Inflection
**PDB ID**	7UX0			
**Wavelength, Å**	0.97946	1.69457	1.13743	1.69515
**Resolution range, Å**	36.01 - 1.50 (1.52 - 1.50)	39.01 - 2.10 (2.14 - 2.10)	29.09 - 1.97 (2.00 - 1.97)	39.02 - 2.12 (2.15 - 2.12)
**Space group**	$P\ 2_1\ 2_1\ 2_1$	$P\ 2_1\ 2_1\ 2_1$	$P\ 2_1\ 2_1\ 2_1$	$P\ 2_1\ 2_1\ 2_1$
**Unit cell**	39.53 43.11 72.02 90 90 90	39.30 43.22 78.01 90 90 90	39.32 43.25 78.09 90 90 90	39.31 43.23 78.03 90 90 90
**Total reflections**	258299 (13130)	90875 (3275)	109780 (6269)	92437 (3364)
**Unique reflections**	20505 (1004)	7810 (370)	8952 (476)	7866 (365)
**Multiplicity**	12.6 (13.1)	11.6 (8.9)	12.3 (13.2)	11.8 (9.2)
**Completeness, %**	99.7 (100.0)	95.7 (92.7)	90.1 (100.0)	98.0 (93.8)
**Mean I/σ(I)**	12.3 (2.2)	14.20 (2.4)	12.10 (2.2)	14.1 (2.1)
R_{merge}	0.148 (1.803)	0.188 (0.922)	0.184 (1.641)	0.173 (1.137)
$CC_{1/2}$	0.997 (0.855)	0.998 (0.882)	0.998 (0.834)	0.998 (0.786)
R_{work}	0.223			
R_{free}	0.251			
**Number of non-hydrogen atoms**	1133			
macromolecules	985			
solvent	89			
**Protein residues**	115			
**RMS(bonds), Å**	0.007			
**RMS(angles), °**	1.03			
**Ramachandran favored, %**	98.20			
**Ramachandran outliers, %**	0.00			
**Clashscore**	3.90			
**Average B-factor**	29.95			
macromolecules	28.69			
solvent	36.35			

Statistics for the highest-resolution shell are shown in parentheses.

Table S2 Summary of the TMEM95 and IZUMO1 monoclonal antibodies

Antigen	Antibody		Isotype	*V*-gene	*D*-gene	*J*-gene	CDR1	CDR2	CDR3
TMEM95	3A01	V_H	IgG1	IGHV5-9-4*01	IGHD1-1*01	IGHJ4*01	NYVMS	EISTYGRYTFYPDSVTG	RDYYGSSSVMDY
		V_L	Kappa	IGKV4-57-1*01		IGKJ1*01	RASSSVSSSSLH	STSNLAS	QQYSGYPLT
	6B08	V_H	IgG1	IGHV5-6*01<br>IGHV5-6-1*01	N/A	IGHJ4*01	TYGMS	TISFYGTHTYYPDILKG	EDYDAMDY
		V_L	Kappa	IGKV9-120*02		IGKJ2*01	RASQDIGSNLN	ATSSLDS	LQYAIFPYT
IZUMO1	4E04	V_H	IgG1	IGHV2-6-5*01	IGHD2-1*01<br>IGHD2-10*01<br>IGHD2-10*02	IGHJ2*01	DFGIS	LIWGGGNTYYNSALKS	HGRFGNTPDY
		V_L	Kappa	IGKV1-110*01		IGKJ1*01	TSGQSLVQSNGNTYLH	KVSNRFS	SQSTRFPWT
	6F02	V_H	IgG1	IGHV3-1*02	IGHD2-4*01<br>IGHD2-9*02	IGHJ2*01	SAYVWH	YIQYSGSTNYNPSLTS	AMITRGYFDY
		V_L	Kappa	IGKV14-111*01		IGKJ4*01	KASQDSNSYLS	GANRLVD	LQYDEFPFT

Table S3 Plasmids and protein sequences used in this study

Plasmids for protein expression from HEK293F transient transfection

Plasmid	Description & encoded protein sequence
pST980	pADD2 Fc
	MGWSCIILFLVATATGVHSENLYFQGGSGGDKTHTCPPCPAPELLGGPSVFLFPPKPKDTLMISRTPEVTCVVVDVSHEDPEVKFNWYVDGVEVHNAKTKPREEQYNSTYRVVSVLTVLHQDWLNGKEYKCKVSNKALPAPIEKTISKAKGQPREPQVYTLPPSRDELTKNQVSLTCLVKGFYPSDIAVEWESNGQPENNYKTTPPVLDSDGSFFLYSKLTVDKSRWQQGNVFSCSVMHEALHNHYTQKSLSLSPGK
pST1392	pADD2 Fc-Avi-His6
	MGWSCIILFLVATATGVHSENLYFQGGSGGDKTHTCPPCPAPELLGGPSVFLFPPKPKDTLMISRTPEVTCVVVDVSHEDPEVKFNWYVDGVEVHNAKTKPREEQYNSTYRVVSVLTVLHQDWLNGKEYKCKVSNKALPAPIEKTISKAKGQPREPQVYTLPPSRDELTKNQVSLTCLVKGFYPSDIAVEWESNGQPENNYKTTPPVLDSDGSFFLYSKLTVDKSRWQQGNVFSCSVMHEALHNHYTQKSLSLSPGKGSGLNDIFEAQKIEWHEGGHHHHHH
pST1359	pADD2 TMEM95-Fc-Avi-His6
	MWRLALGGVFLAAAQACVFCRLPAHDLSGRLARLCSQMEARQKECGASPDFSAFALDEVSMNKVTEKTHRVLRVMEIKEAVSSLPSYWSWLRKTKLPEYTREALCPPACRGSTTLYNCSTCKGTEVSCWPRKRCFPGSQDLWEAKENLYFQGGSGGDKTHTCPPCPAPELLGGPSVFLFPPKPKDTLMISRTPEVTCVVVDVSHEDPEVKFNWYVDGVEVHNAKTKPREEQYNSTYRVVSVLTVLHQDWLNGKEYKCKVSNKALPAPIEKTISKAKGQPREPQVYTLPPSRDELTKNQVSLTCLVKGFYPSDIAVEWESNGQPENNYKTTPPVLDSDGSFFLYSKLTVDKSRWQQGNVFSCSVMHEALHNHYTQKSLSLSPGKGSGLNDIFEAQKIEWHEGGHHHHHH
pST1094	pADD2 IZUMO1-Fc
	MGPHFTLLCAALAGCLLPAEGCVICDPSVVLALKSLEKDYLPGHLDAKHHKAMMERVENAVKDFQELSLNEDAYMGVVDEATLQKGSWSLLKDLKRITDSDVKGDLFVKELFWMLHLQKETFATYVARFQKEAYCPNKCGVMLQTLIWCKNCKKEVHACRKSYDCGERNVEVPQMEDMILDCELNWHQASEGLTDYSFYRVWGNNTETLVSKGKEATLTKPMVGPEDAGSYRCELGSVNSSPATIINFHVTVLPKENLYFQGGSGGDKTHTCPPCPAPELLGGPSVFLFPPKPKDTLMISRTPEVTCVVVDVSHEDPEVKFNWYVDGVEVHNAKTKPREEQYNSTYRVVSVLTVLHQDWLNGKEYKCKVSNKALPAPIEKTISKAKGQPREPQVYTLPPSRDELTKNQVSLTCLVKGFYPSDIAVEWESNGQPENNYKTTPPVLDSDGSFFLYSKLTVDKSRWQQGNVFSCSVMHEALHNHYTQKSLSLSPGK
pST1373	pADD2 IZUMO1-Fc-Avi-His6
	MGPHFTLLCAALAGCLLPAEGCVICDPSVVLALKSLEKDYLPGHLDAKHHKAMMERVENAVKDFQELSLNEDAYMGVVDEATLQKGSWSLLKDLKRITDSDVKGDLFVKELFWMLHLQKETFATYVARFQKEAYCPNKCGVMLQTLIWCKNCKKEVHACRKSYDCGERNVEVPQMEDMILDCELNWHQASEGLTDYSFYRVWGNNTETLVSKGKEATLTKPMVGPEDAGSYRCELGSVNSSPATIINFHVTVLPKENLYFQGGSGGDKTHTCPPCPAPELLGGPSVFLFPPKPKDTLMISRTPEVTCVVVDVSHEDPEVKFNWYVDGVEVHNAKTKPREEQYNSTYRVVSVLTVLHQDWLNGKEYKCKVSNKALPAPIEKTISKAKGQPREPQVYTLPPSRDELTKNQVSLTCLVKGFYPSDIAVEWESNGQPENNYKTTPPVLDSDGSFFLYSKLTVDKSRWQQGNVFSCSVMHEALHNHYTQKSLSLSPGKGSGLNDIFEAQKIEWHEGGHHHHHH
pST1710	pADD2 IZUMO1-Fc-Avi-His6 W148A
	MGPHFTLLCAALAGCLLPAEGCVICDPSVVLALKSLEKDYLPGHLDAKHHKAMMERVENAVKDFQELSLNEDAYMGVVDEATLQKGSWSLLKDLKRITDSDVKGDLFVKELFWMLHLQKETFATYVARFQKEAYCPNKCGVMLQTLIACKNCKKEVHACRKSYDCGERNVEVPQMEDMILDCELNWHQASEGLTDYSFYRVWGNNTETLVSKGKEATLTKPMVGPEDAGSYRCELGSVNSSPATIINFHVTVLPKENLYFQGGSGGDKTHTCPPCPAPELLGGPSVFLFPPKPKDTLMISRTPEVTCVVVDVSHEDPEVKFNWYVDGVEVHNAKTKPREEQYNSTYRVVSVLTVLHQDWLNGKEYKCKVSNKALPAPIEKTISKAKGQPREPQVYTLPPSRDELTKNQVSLTCLVKGFYPSDIAVEWESNGQPENNYKTTPPVLDSDGSFFLYSKLTVDKSRWQQGNVFSCSVMHEALHNHYTQKSLSLSPGKGSGLNDIFEAQKIEWHEGGHHHHHH
pST1557	pADD2 TMEM95-Ctag
	MRMQLLLLIALSLALVTNSCVFCRLPAHDLSGRLARLCSQMEARQKECGASPDFSAFALDEVSMNKVTEKTHRVLRVMEIKEAVSSLPSYWSWLRKTKLPEYTREALCPPACRGSTTLYNCSTCKGTEVSCWPRKRCFPGSEPEA

pST1704	pADD2 TMEM95-Fc-Avi-His6 R70A
	MWRLALGGVFLAAAQACVFCRLPAHDLSGRLARLCSQMEARQKECGASPDFSAFALDEVSMNKVTEKTHA VLRVMEIKEAVSSLPSYWSWLRKTKLPEYTREALCPPACRGSTTLYNCSTCKGTEVSCWPRKRCFPGSQD LWEAKENLYFQGGSGGDKTHTCPPCPAPELLGGPSVFLFPPKPKDTLMISRTPEVTCVVVDVSHEDPEVK FNWYVDGVEVHNAKTKPREEQYNSTYRVVSVLTVLHQDWLNGKEYKCKVSNKALPAPIEKTISKAKGQPR EPQVYTLPPSRDELTKNQVSLTCLVKGFYPSDIAVEWESNGQPENNYKTTPPVLDSDGSFFLYSKLTVDK SRWQQGNVFSCSVMHEALHNHYTQKSLSLSPGKGSGLNDIFEAQKIEWHEGGHHHHHH
pST1705	pADD2 TMEM95-Fc-Avi-His6 R73A
	MWRLALGGVFLAAAQACVFCRLPAHDLSGRLARLCSQMEARQKECGASPDFSAFALDEVSMNKVTEKTHR VLAVMEIKEAVSSLPSYWSWLRKTKLPEYTREALCPPACRGSTTLYNCSTCKGTEVSCWPRKRCFPGSQD LWEAKENLYFQGGSGGDKTHTCPPCPAPELLGGPSVFLFPPKPKDTLMISRTPEVTCVVVDVSHEDPEVK FNWYVDGVEVHNAKTKPREEQYNSTYRVVSVLTVLHQDWLNGKEYKCKVSNKALPAPIEKTISKAKGQPR EPQVYTLPPSRDELTKNQVSLTCLVKGFYPSDIAVEWESNGQPENNYKTTPPVLDSDGSFFLYSKLTVDK SRWQQGNVFSCSVMHEALHNHYTQKSLSLSPGKGSGLNDIFEAQKIEWHEGGHHHHHH
pST1761	pADD2 TMEM95-Fc-Avi-His6 R70A R73A
	MWRLALGGVFLAAAQACVFCRLPAHDLSGRLARLCSQMEARQKECGASPDFSAFALDEVSMNKVTEKTHA VLAVMEIKEAVSSLPSYWSWLRKTKLPEYTREALCPPACRGSTTLYNCSTCKGTEVSCWPRKRCFPGSQD LWEAKENLYFQGGSGGDKTHTCPPCPAPELLGGPSVFLFPPKPKDTLMISRTPEVTCVVVDVSHEDPEVK FNWYVDGVEVHNAKTKPREEQYNSTYRVVSVLTVLHQDWLNGKEYKCKVSNKALPAPIEKTISKAKGQPR EPQVYTLPPSRDELTKNQVSLTCLVKGFYPSDIAVEWESNGQPENNYKTTPPVLDSDGSFFLYSKLTVDK SRWQQGNVFSCSVMHEALHNHYTQKSLSLSPGKGSGLNDIFEAQKIEWHEGGHHHHHH
pST1720	pVRC anti-TMEM95 3A01 IgG1 heavy chain
	MGWSCIILFLVATATGVHSEVQLVESGGDLVRPGGSLKLSCVVSGFAFSNYVMSWVRQSPEKRLEWVAEI STYGRYTFYPDSVTGRFTISRDNAKNTLFLEMSSLRSEDSAMYYCARRDYYGSSSVMDYWGQGTSVIVSS AKTTPPSVYPLAPGSAAQTNSMVTLGCLVKGYFPEPVTVTWNSGSLSSGVHTFPAVLQSDLYTLSSSVTV PSSTWPSETVTCNVAHPASSTKVDKKIVPRDCGCKPCICTVPEVSSVFIFPPKPKDVLTITLTPKVTCVV VDISKDDPEVQFSWFVDDVEVHTAQTQPREEQFNSTFRSVSELPIMHQDWLNGKEFKCRVNSAAFPAPIE KTISKTKGRPKAPQVYTIPPPKEQMAKDKVSLTCMITDFFPEDITVEWQWNGQPAENYKNTQPIMDTDGS YFVYSKLNVQKSNWEAGNTFTCSVLHEGLHNHHTEKSLSHSPGK
pST1721	pVRC anti-TMEM95 3A01 Fab heavy chain
	MGWSCIILFLVATATGVHSEVQLVESGGDLVRPGGSLKLSCVVSGFAFSNYVMSWVRQSPEKRLEWVAEI STYGRYTFYPDSVTGRFTISRDNAKNTLFLEMSSLRSEDSAMYYCARRDYYGSSSVMDYWGQGTSVIVSS AKTTPPSVYPLAPGSAAQTNSMVTLGCLVKGYFPEPVTVTWNSGSLSSGVHTFPAVLQSDLYTLSSSVTV PSSTWPSETVTCNVAHPASSTKVDKKIVPRDC
pST1722	pVRC anti-TMEM95 3A01 light chain
	MGWSCIILFLVATATGVHSENVLTQSPAIMSASPGEKVTMPCRASSSVSSSSLHWYQQKSGASPKLWIYS TSNLASGVPARFSGSGSGTSYSLTITSVEAEDAATYYCQQYSGYPLTFGGGTKLEIKADAAPTVSIFPPS SEQLTSGGASVVCFLNNFYPKDINVKWKIDGSERQNGVLNSWTDQDSKDSTYSMSSTLTLTKDEYERHNS YTCEATHKTSTSPIVKSFNRNEC
pST1723	pVRC anti-TMEM95 6B08 IgG1 heavy chain
	MGWSCIILFLVATATGVHSEVQLVESGGDLVKPGGSLKLSCAASGFTFSTYGMSWVRQTPDKRLEWVATI SFYGTHTYYPDILKGRFTISRENAKNTLYLQMSSLKSEDTAMYFCAREDYDAMDYWGQGTSVTVSSAKTT PPSVYPLAPGSAAQTNSMVTLGCLVKGYFPEPVTVTWNSGSLSSGVHTFPAVLQSDLYTLSSSVTVPSST WPSETVTCNVAHPASSTKVDKKIVPRDCGCKPCICTVPEVSSVFIFPPKPKDVLTITLTPKVTCVVVDIS KDDPEVQFSWFVDDVEVHTAQTQPREEQFNSTFRSVSELPIMHQDWLNGKEFKCRVNSAAFPAPIEKTIS KTKGRPKAPQVYTIPPPKEQMAKDKVSLTCMITDFFPEDITVEWQWNGQPAENYKNTQPIMDTDGSYFVY SKLNVQKSNWEAGNTFTCSVLHEGLHNHHTEKSLSHSPGK
pST1724	pVRC anti-TMEM95 6B08 Fab heavy chain
	MGWSCIILFLVATATGVHSEVQLVESGGDLVKPGGSLKLSCAASGFTFSTYGMSWVRQTPDKRLEWVATI SFYGTHTYYPDILKGRFTISRENAKNTLYLQMSSLKSEDTAMYFCAREDYDAMDYWGQGTSVTVSSAKTT PPSVYPLAPGSAAQTNSMVTLGCLVKGYFPEPVTVTWNSGSLSSGVHTFPAVLQSDLYTLSSSVTVPSST WPSETVTCNVAHPASSTKVDKKIVPRDC
pST1725	pVRC anti-TMEM95 6B08 light chain
	MGWSCIILFLVATATGVHSDIQMTQSPSSLSASLGERVSLTCRASQDIGSNLNWLQQEPDGTIKRLIYAT SSLDSGVPKRFSGSRSGSDYSLTISSLESEDFVDYYCLQYAIFPYTFGGGTKLEIKADAAPTVSIFPPSS

	EQLTSGGASVVCFLNNFYPKDINVKWKIDGSERQNGVLNSWTDQDSKDSTYSMSSTLTLTKDEYERHNSY<br>TCEATHKTSTSPIVKSFNRNEC
pST1776	pVRC anti-IZUMO1 4E04 IgG1 heavy chain
	MGWSCIILFLVATATGVHSQVQLKESGPGLVAPSQSLSITCTVSGFSLTDFGISWIRQPPGKGLEWLGLI<br>WGGGNTYYNSALKSRLSISKDNSKSQVFLKMNSLQTDDTAMYYCAKHGRFGNTPDYWGQGTTLTVSSAKT<br>TPPSVYPLAPGSAAQTNSMVTLGCLVKGYFPEPVTVTWNSGSLSSGVHTFPAVLQSDLYTLSSSVTVPSS<br>TWPSETVTCNVAHPASSTKVDKKIVPRDCGCKPCICTVPEVSSVFIFPPKPKDVLTITLTPKVTCVVVDI<br>SKDDPEVQFSWFVDDVEVHTAQTQPREEQFNSTFRSVSELPIMHQDWLNGKEFKCRVNSAAFPAPIEKTI<br>SKTKGRPKAPQVYTIPPPKEQMAKDKVSLTCMITDFFPEDITVEWQWNGQPAENYKNTQPIMDTDGSYFV<br>YSKLNVQKSNWEAGNTFTCSVLHEGLHNHHTEKSLSHSPGK
pST1777	pVRC anti-IZUMO1 4E04 Fab heavy chain
	MGWSCIILFLVATATGVHSQVQLKESGPGLVAPSQSLSITCTVSGFSLTDFGISWIRQPPGKGLEWLGLI<br>WGGGNTYYNSALKSRLSISKDNSKSQVFLKMNSLQTDDTAMYYCAKHGRFGNTPDYWGQGTTLTVSSAKT<br>TPPSVYPLAPGSAAQTNSMVTLGCLVKGYFPEPVTVTWNSGSLSSGVHTFPAVLQSDLYTLSSSVTVPSS<br>TWPSETVTCNVAHPASSTKVDKKIVPRDC
pST1778	pVRC anti-IZUMO1 4E04 light chain
	MGWSCIILFLVATATGVHSDVVMTQTPLSLPVSLGDQASFSCTSGQSLVQSNGNTYLHWYLQKPGQSPKL<br>LIYKVSNRFSGVPDRFSGSGSGTDFTLKISRVEAEDLGVYFCSQSTRFPWTFGGGTKLEIKADAAPTVSI<br>FPPSSEQLTSGGASVVCFLNNFYPKDINVKWKIDGSERQNGVLNSWTDQDSKDSTYSMSSTLTLTKDEYE<br>RHNSYTCEATHKTSTSPIVKSFNRNEC
pST1779	pVRC anti-IZUMO1 6F02 IgG1 heavy chain
	MGWSCIILFLVATATGVHSDVQLQESGPDLVKPSQSPSLTCTVTGYSITSAYVWHWIRQFPGNKLEWMGY<br>IQYSGSTNYNPSLTSRISITRDTSKNQFFLKLKSVTTADTATYYCARAMITRGYFDYWGQGTTLTVSSAK<br>TTPPSVYPLAPGSAAQTNSMVTLGCLVKGYFPEPVTVTWNSGSLSSGVHTFPAVLQSDLYTLSSSVTVPS<br>STWPSETVTCNVAHPASSTKVDKKIVPRDCGCKPCICTVPEVSSVFIFPPKPKDVLTITLTPKVTCVVVD<br>ISKDDPEVQFSWFVDDVEVHTAQTQPREEQFNSTFRSVSELPIMHQDWLNGKEFKCRVNSAAFPAPIEKT<br>ISKTKGRPKAPQVYTIPPPKEQMAKDKVSLTCMITDFFPEDITVEWQWNGQPAENYKNTQPIMDTDGSYF<br>VYSKLNVQKSNWEAGNTFTCSVLHEGLHNHHTEKSLSHSPGK
pST1780	pVRC anti-IZUMO1 6F02 Fab heavy chain
	MGWSCIILFLVATATGVHSDVQLQESGPDLVKPSQSPSLTCTVTGYSITSAYVWHWIRQFPGNKLEWMGY<br>IQYSGSTNYNPSLTSRISITRDTSKNQFFLKLKSVTTADTATYYCARAMITRGYFDYWGQGTTLTVSSAK<br>TTPPSVYPLAPGSAAQTNSMVTLGCLVKGYFPEPVTVTWNSGSLSSGVHTFPAVLQSDLYTLSSSVTVPS<br>STWPSETVTCNVAHPASSTKVDKKIVPRDC
pST1783	pVRC anti-IZUMO1 6F02 light chain
	MGWSCIILFLVATATGVHSDIKMTQSPSSMYASLGERVTITCKASQDSNSYLSWIQQKPGKSPKTLIYGA<br>NRLVDGVPSRFSGSGSGQDYSLTISSLEYEDMGFYYCLQYDEFPFTFGSGTKLETKADAAPTVSIFPPSS<br>EQLTSGGASVVCFLNNFYPKDINVKWKIDGSERQNGVLNSWTDQDSKDSTYSMSSTLTLTKDEYERHNSY<br>TCEATHKTSTSPIVKSFNRNEC

Plasmid for protein expression from baculovirus infection

Plasmid	Description & encoded protein sequence
pST1618	pACgp67a JUNO-His6
	MVSAIVLYVLLAAAAHSAFAGDELLNICMNAKHHKRVPSPEDKLYEECIPWKDNACCTLTTSWEAHLDVS<br>PLYNFSLFHCGLLMPGCRKHFIQAICFYECSPNLGPWIQPVGSLGWEVAPSGQGERVVNVPLCQEDCEEW<br>WEDCRMSYTCKSNWRGGWDWSQGKNRCPKGAQCLPFSHYFPTPADLCEKTWSNSFKASPERRNSGRCLQK<br>WFEPAQGNPNVAVARLFAGGHHHHHH

SI Reference

1. Y. Tsunoda, M. C. Chang, Further studies of antisera on the fertilization of mouse, rat, and hamster eggs in vivo and in vitro. *Int J Fertil* **22**, 129-139 (1977).

2. C. Vonrhein *et al.*, Data processing and analysis with the autoPROC toolbox. *Acta crystallographica. Section D, Biological crystallography* **67**, 293-302 (2011).

3. D. Liebschner *et al.*, Macromolecular structure determination using X-rays, neutrons and electrons: recent developments in Phenix. *Acta Crystallogr D Struct Biol* **75**, 861-877 (2019).

4. P. Emsley, K. Cowtan, Coot: model-building tools for molecular graphics. *Acta crystallographica. Section D, Biological crystallography* **60**, 2126-2132 (2004).

5. H. Ashkenazy *et al.*, ConSurf 2016: an improved methodology to estimate and visualize evolutionary conservation in macromolecules. *Nucleic acids research* **44**, W344-350 (2016).

6. W. M. Skinner, N. Mannowetz, P. V. Lishko, N. R. Roan, Single-cell Motility Analysis of Tethered Human Spermatozoa. *Bio Protoc* **9** (2019).
